# Stable Ultramicroporous Metal–Organic Framework with Hydrophilic and Hydrophobic Domains for Selective Gas Adsorption

**DOI:** 10.1002/anie.202513788

**Published:** 2025-08-23

**Authors:** Robert Oestreich, Marcus N. A. Fetzer, Yifei Zhang, Andreas Schreiber, Alexander Knebel, Markus Suta, Christoph Janiak, Gabriel Hanna, Gündoğ Yücesan

**Affiliations:** ^1^ Institute for Inorganic and Structural Chemistry Heinrich Heine University Düsseldorf Universitätsstr. 1 D‐40225 Düsseldorf Germany; ^2^ Inorganic Photoactive Materials, Institute for Inorganic and Structural Chemistry Heinrich Heine University Düsseldorf Universitätsstr. 1 40225 Düsseldorf Germany; ^3^ Microtrac Retsch GmbH Retsch‐Allee 1–5 D‐42781 Haan Germany; ^4^ Otto Schott Institute of Materials Research, Center for Energy and Environmental Chemistry II Friedrich Schiller University Jena Lessingstraße 12–14 D‐07743 Jena Germany; ^5^ Center for Energy and Environmental Chemistry Friedrich Schiller University Jena Philosophenweg 7a D‐07743 Jena Germany; ^6^ Department of Chemistry University of Alberta Edmonton Alberta Canada

**Keywords:** Chemically & thermally stable MOFs, CO_2_ capture, Gas separation

## Abstract

Herein, we report the thermal and chemical stability, and the gas adsorption behavior, of a mixed‐linker phosphonate MOF, [Cu(4,4′‐bpy)_0.5_(1,4‐NDPAH_2_)], namely TUB41 (where bpy = bipyridine and NDPAH_4_ = naphthalenediphosphonic acid). TUB41 demonstrates remarkable chemical stability across a wide pH range (1–11) and retains its structural integrity after 2 years of repeated adsorption cycles and activation at 80 °C under ambient humidity. Cryogenic adsorption experiments reveal that TUB41's pores selectively exclude gases with larger kinetic diameters, such as N_2_ and Ar, while accommodating smaller molecules like CO_2_ and H_2_O at elevated temperatures. The enthalpies of adsorption for CO_2_ at a loading 0.01 mmol g^−1^ and H_2_O at a loading of 0.7 mmol g^−1^ are −41 and −38 kJ mol^−1^, respectively, reflecting their strongly attractive interactions with TUB41 under different conditions. Molecular dynamics simulations reveal that CO_2_ molecules adopt ordered arrangements in the central hydrophobic regions of the pores, guided by strong nonbonding interactions, while H_2_O molecules preferentially bind to the hydrophilic secondary building units. Mean‐squared displacement analyses confirm that both gases remain spatially constrained within the pores. These findings highlight TUB41 as a chemically robust and highly selective MOF, with potential for applications in gas separation, photocatalytic water splitting, and CO_2_ reduction under challenging conditions.

## Graphical abstract

The ultramicroporous pores of TUB41 selectively host water and CO_2_ molecules at distinct hydrophilic and hydrophobic compartments without competition.

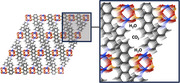

## Introduction

Metal–organic frameworks (MOFs) have evolved as one of the most studied material classes with a wide range of potential industrial applications in medicine, food chemistry, catalysis, energy storage, and many others.^[^ ^1^, ^2^, ^3^, ^4^ ^]^ For each application, MOFs must possess unique chemical and thermal stabilities suitable for the desired application. For example, biodegradability is an essential requirement for MOFs to be used for applications such as drug delivery,^[^ ^5^, ^6^, ^7^, ^8^, ^9^ ^]^ while MOFs intended for use in batteries, supercapacitors, electrocatalysis (including hydrogen evolution and oxygen evolution reactions), CO_2_ capture, and water harvesting must demonstrate sufficient stability to survive in the presence of electrolytes, water, and acidic or basic environments.^[^ ^10^ ^]^ Despite the rich structural diversity of MOFs and their vast potential applications,^[^ ^2^ ^]^ there still is only a handful of MOFs in the literature, which are considered to be stable in the presence of water, acids, bases, and electrolytes.^[^ ^11^, ^12^ ^]^ Notably, UiO‐66, which is known for its exceptional stability, has a shelf lifetime of ca. 2 months at room temperature and ambient humidity.^[^ ^13^, ^14^ ^]^ Therefore, the development of highly stable MOFs would finally open the way towards the wide‐spread industrial use of MOFs. The secondary building units (SBUs) of conventional carboxylate MOFs are usually composed of water‐labile metal carboxylate clusters, including Zr(IV) carboxylate clusters (as in UiO‐66), which limit conventional MOF applications in aqueous media.^[^ ^14^ ^]^ Furthermore, the CO_2_ adsorption process also generates an acidic environment, which is challenging for conventional MOFs to survive for a long period of time.^[^ ^15^ ^]^ Phosphonic acid‐based MOFs provide a promising route to achieving stability in acidic media.^[^ ^16^ ^]^ Compared to conventional metal‐binding functional groups, phosphonic acids are very robust organic linkers that survive in the presence of concentrated acids, and the P─C bond is known to be stable in the presence of UV‐light and at high temperatures.^[^ ^17^, ^18^ ^]^ The formation of insoluble metal phosphonate SBUs is advantageous because it enhances the structural stability of the MOF in aqueous environments, unlike metal acetate SBUs, which are typically water‐soluble and prone to degradation.^[^ ^17^, ^19^, ^20^ ^]^

While significant progress has been made in developing amine‐functionalized MOFs for CO_2_ capture via chemisorption (where CO_2_ forms covalent bonds with amine groups), physisorptive capture remains a greater challenge.^[^ ^15^ ^]^ This is largely due to the intrinsic hydrophilicity of many MOFs, which are often constructed from organic linkers bearing polar functional groups and SBUs that interact favorably with water vapor.^[^ ^21^ ^]^ Although physisorption is technically simpler and more energy‐efficient than chemisorption, especially when employing pressure swing adsorption, achieving selective CO_2_ uptake in the presence of humidity has proven difficult.^[^ ^22^ ^]^ Only a few MOFs have been reported to exhibit CO_2_ selectivity under humid conditions.^[^ ^15^ ^]^ Among them, CALF‐20 shows promising performance at 10% RH but loses selectivity entirely at 40% RH.^[^ ^15^ ^]^ Moreover, its chemical stability was tested only under gas‐phase conditions in the original study. While amine‐based systems and frameworks like MOF‐74 demonstrate high CO_2_ capacities, their chemical instability under humid or acidic/basic aqueous conditions severely limits practical applications.^[^ ^23^, ^24^, ^25^, ^26^, ^27^ ^]^ Some well‐studied MOFs, such as MOF‐74 and DEF‐2, decompose rapidly in air, and others lack reported stability data altogether.^[^ ^23^, ^25^ ^]^ Most chemical stability tests for CO_2_‐adsorbing MOFs rely on repeated adsorption‐desorption cycles. As summarized in several review articles, only a few MOFs have demonstrated exceptional long‐term stability in various chemical media. Notable examples include MIL‐100(Cr), which remains stable in water for up to one year; Ni(BTP)_2_, which retains its structural integrity for 14 days across a wide pH range (2 to 14); and ZIF‐8, which is stable for 7 days in boiling organic solvents and water, and for 24 h in boiling NaOH.^[^ ^11^, ^28^ ^]^ One approach to overcoming this limitation involves designing chemically stable MOFs with unconventional organic linkers containing phosphonic acid functional groups.^[^ ^29^, ^30^, ^31^ ^]^ These groups form strong coordination bonds with metal ions, leading to the formation of water‐insoluble metal phosphonate SBUs that are highly resistant to hydrolysis in acidic/basic media.^[^ ^29^, ^31^ ^]^ Such MOFs offer a promising route towards physisorptive CO_2_ capture that remain stable and selective in humid environments.

Our group and others have reported the stability of phosphonate MOFs in harsh environments, including strong acids, bases, and even aqua regia, making them highly promising candidates for diverse applications such as electrocatalysis, energy storage, and sequestration of greenhouse gases.^[^ ^17^, ^30^, ^31^, ^32^ ^]^ Additionally, our investigations into hydrogen‐bonded organic frameworks (HOFs) and polyphosphonate covalent organic frameworks (COFs) constructed from phosphonic acids have shown exceptional stability in humid environments during and after proton conductivity experiments.^[^ ^33^, ^34^, ^35^, ^36^ ^]^ Although the short‐term chemical stability of phosphonate MOFs is well‐documented, their long‐term stability over extended periods has, to the best of our knowledge, not been reported in the literature. It has been also shown that metal phosphonates might be electrically conductive.^[^ ^4^, ^37^, ^38^ ^]^ Despite their inherent chemical stability and promising potential for applications in gas storage, energy storage, and catalysis, phosphonate MOFs remain relatively underexplored. Their development is largely hindered by the limited commercial availability of arylphosphonic acid linkers and fundamental phosphonate precursors.^[^ ^29^ ^]^ Overcoming these challenges is essential for advancing the design of sustainable MOF families capable of operating in harsh chemical environments.

**Figure 1:**
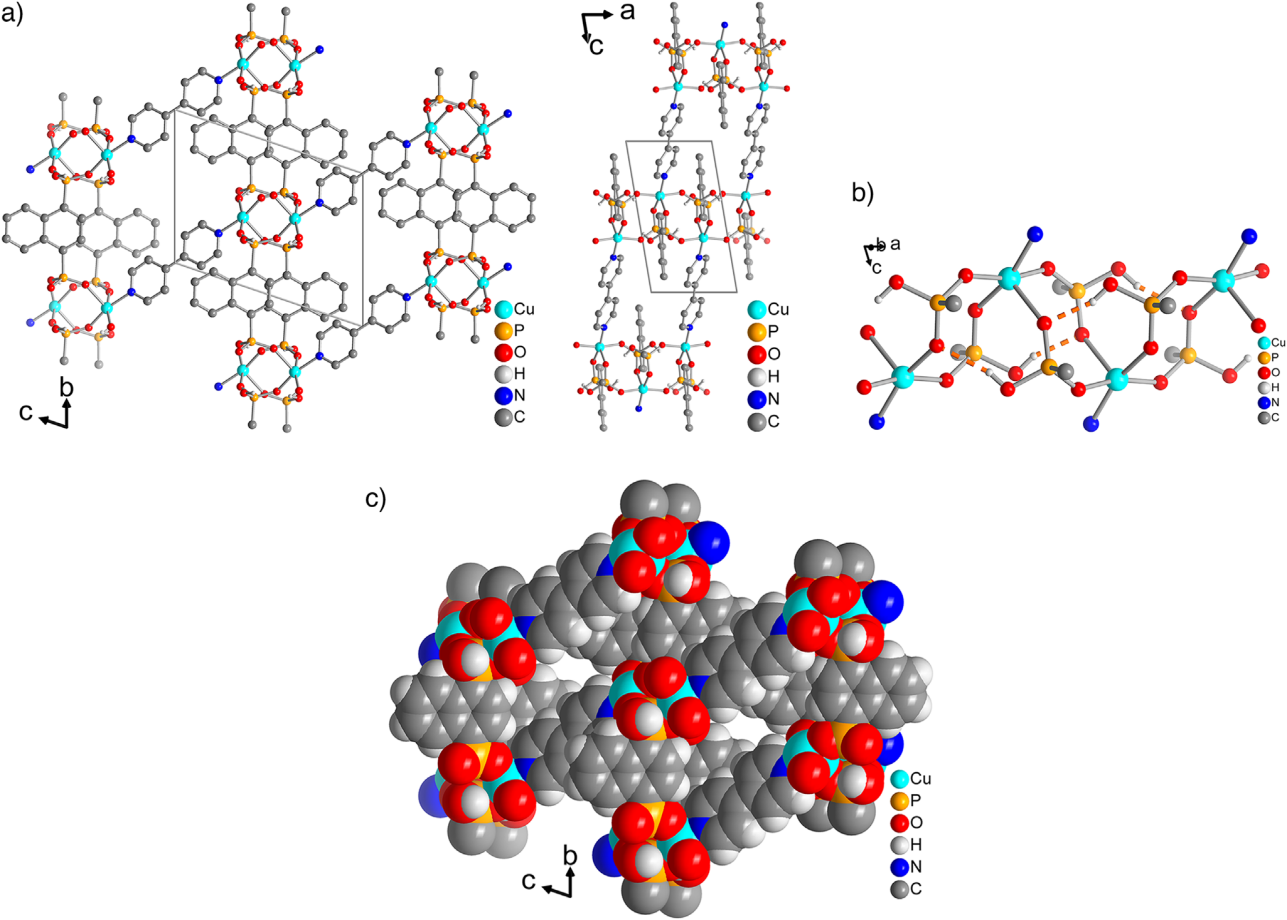
a) Section of the packing in the crystal structure of TUB41 along two different viewing directions (hydrogen atoms on carbon are omitted for clarity). b) SBU of TUB41 running along the *a* direction, showing the hydrogen bonds between the phosphonate groups. c) Space‐filling representation to visualize the small ∼4 × 2 Å^2^ cross section of the slit‐shaped channels along the *a* axis (cf. Figure S18 for a grid scale).

In this work, we focus on naphthalene as a linker core, which is a hydrophobic and polyaromatic moiety to create MOFs that could capture CO_2_.^[^ ^33^ ^]^ Previously, we published a structural report on TUB41, a mixed‐linker MOF utilizing 1,4‐naphthalenediphosphonic acid (1,4‐NDPAH_4_) and 4,4′‐bipyridine (4,4′‐bpy) (see Figure 1a).^[^ ^39^ ^]^ However, due to its very low yield, we were not able to work on its applications previously. In this work, we synthesized TUB41 hydrothermally as a single product phase in high yield, and thus were able to study its chemical/thermal stability. Furthermore, since TUB41 has narrow and largely hydrophobic pores, which are suitable for sequestering molecules with small kinetic diameters (e.g., CO_2_ and H_2_O), we studied its ability to capture CO_2_ and H_2_O. Notably, TUB41 was found to have a shelf lifetime of beyond 2 years after repeated gas adsorption experiments and activation at 80 °C under vacuum, which is a major improvement over the 2 months for conventional carboxylate‐based MOFs such as UiO‐66.

In more detail, as shown in Figure 1b, TUB41 has a rod‐shaped SBU composed of typical eight‐membered Cu‐O‐P‐O‐Cu‐O‐P‐O rings observed in phosphonate MOFs, which are bridged by the square pyramidal Cu(II) atoms. The hydrogen bonding between the Cu(II)‐coordinating phosphonate groups defines the final shape of the SBU and provides a hydrophilic character to the SBU. The hydrophilic SBUs with hydrogen bonds are surrounded by hydrophobic naphthalene moieties, hypothetically limiting the noncovalent interactions of the SBU with hydrophilic gases and generating a more preferable environment for gases such as CO_2_. Recently, MOF research has focused on frameworks with narrow pores to enhance gas adsorption selectivity.^[^ ^40^ ^]^ The combination of narrow pores and a pronounced hydrophobicity in TUB41, along with its thermal and chemical stability, makes it highly desirable for the development of industrial MOF applications that can selectively capture or separate gases such as CO_2_ in the presence of H_2_O.^[^ ^41^, ^42^ ^]^

## Chemical Stability of TUB41

**Figure 2:**
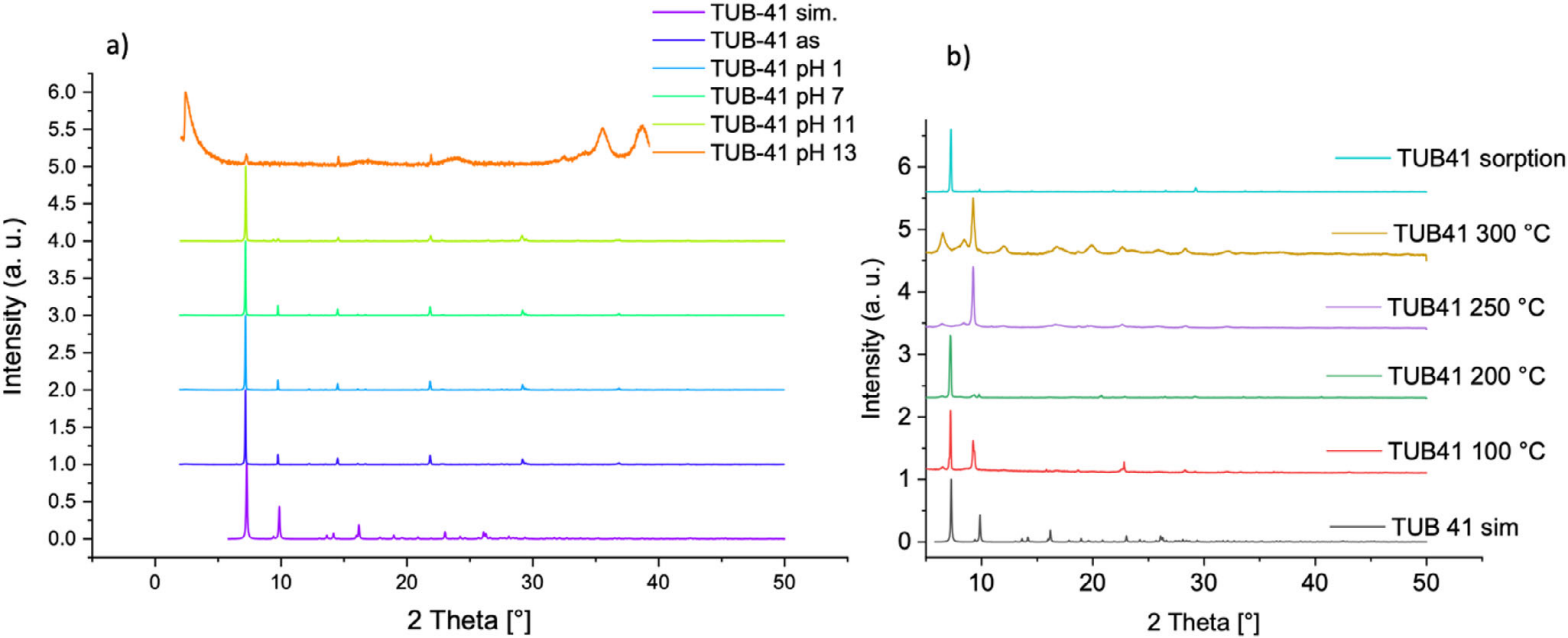
PXRD (Cu *K_α_* radiation) of TUB41 at a) different pH's and b) different temperatures after adsorption experiments.

**Figure 3:**
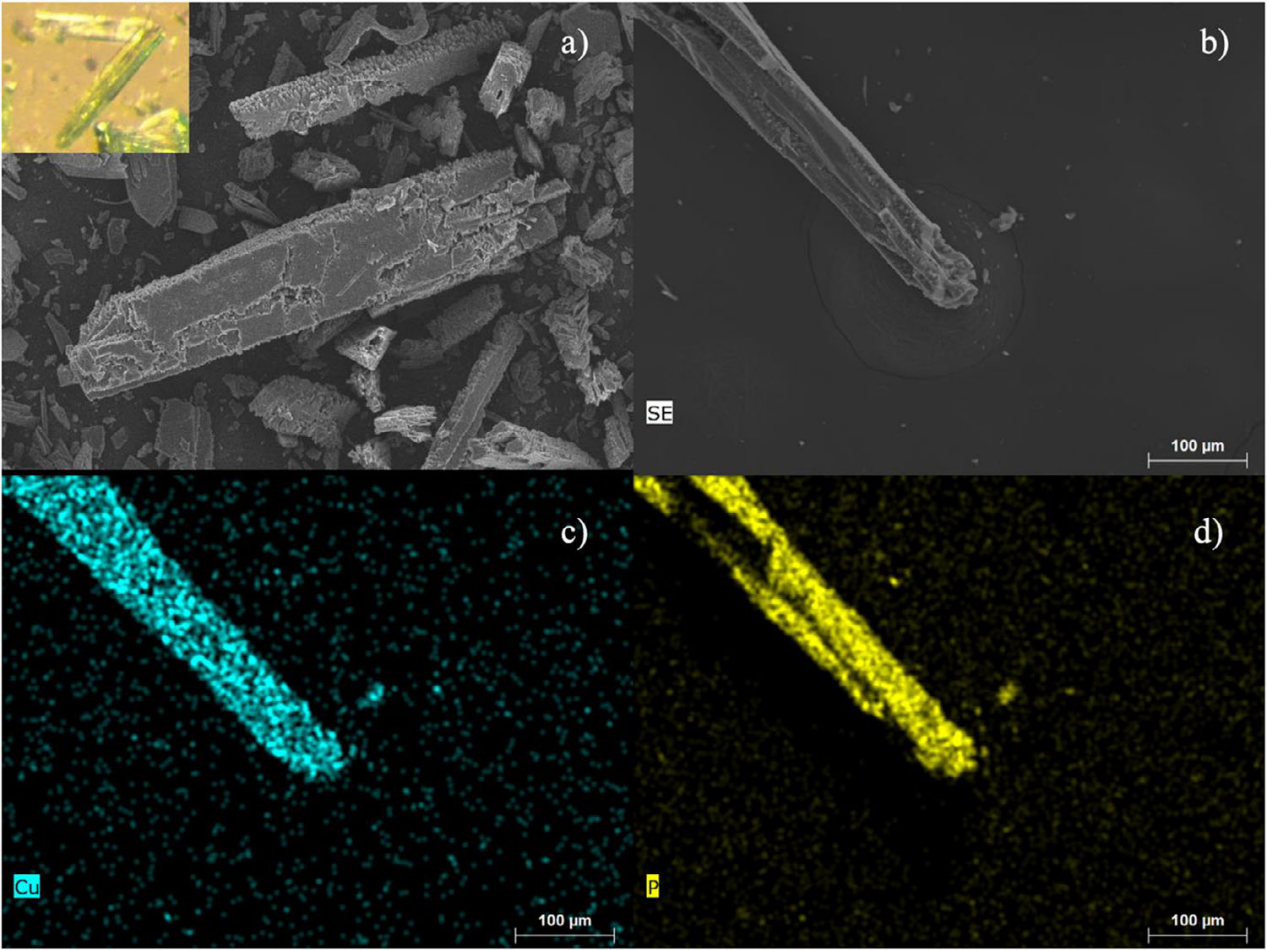
SEM pictures of TUB41 after adsorption experiments. Crystals of TUB41 are shown in panels a) and b), and the corresponding elemental mappings for Cu and P are shown in panels c) and d), respectively.

The crystals of TUB41 were reproduced in Fall 2022. Chemical stability tests on TUB41 were performed by suspending TUB41 crystals in aqueous HCl and NaOH solutions ranging in pH between 1 and 13 for 2 h. In addition, TUB41 samples were left in the hydrothermal reaction mixture (an aqueous medium of pH 2.5) for ca. 1 month before the purification of the crystals, pointing to its stability. As depicted in Figure 2a, the powder X‐ray diffraction (PXRD) patterns of the acid‐ and base‐treated samples of TUB41 retain their structures between pH 1 and pH 11 after 2 h, highlighting its stability in acidic and basic environments for this period of time. The sample treated at pH 13 starts to decompose, although it retains some of its original PXRD pattern. At this pH, it predominantly converts into CuOH species, indicating relatively slow decomposition. In addition, TUB41 was synthesized in Parr acid digestion reaction vessels in distilled water at pH 1.5 (pH 2.5 after the reaction) and 120 °C for 48 h, which also points to the intrinsic chemical stability of TUB41 in an acidic environment. As shown in Figure 3, the SEM images reveal that the crystals largely retain their morphology after 2 years of repeated adsorption experiments and storage under room temperature and ambient humidity conditions.

## Thermal Stability of TUB41

To understand the thermal behavior of TUB41, we initially performed PXRD of samples that were heated up to 300 °C for 1 h periods, and collected the PXRD data after cooling them to room temperature (Figure 2b). Phosphonic acids condense at high temperatures to make P─O─P bonds, potentially leading to a crystalline metal polymeric framework at ca. 300 °C.^[^ ^36^ ^]^ This is suggested by the MS‐TGA results, which show that water evaporation gradually occurs above ca. 250 °C (see Figure 5 and Section 4).

**Figure 4:**
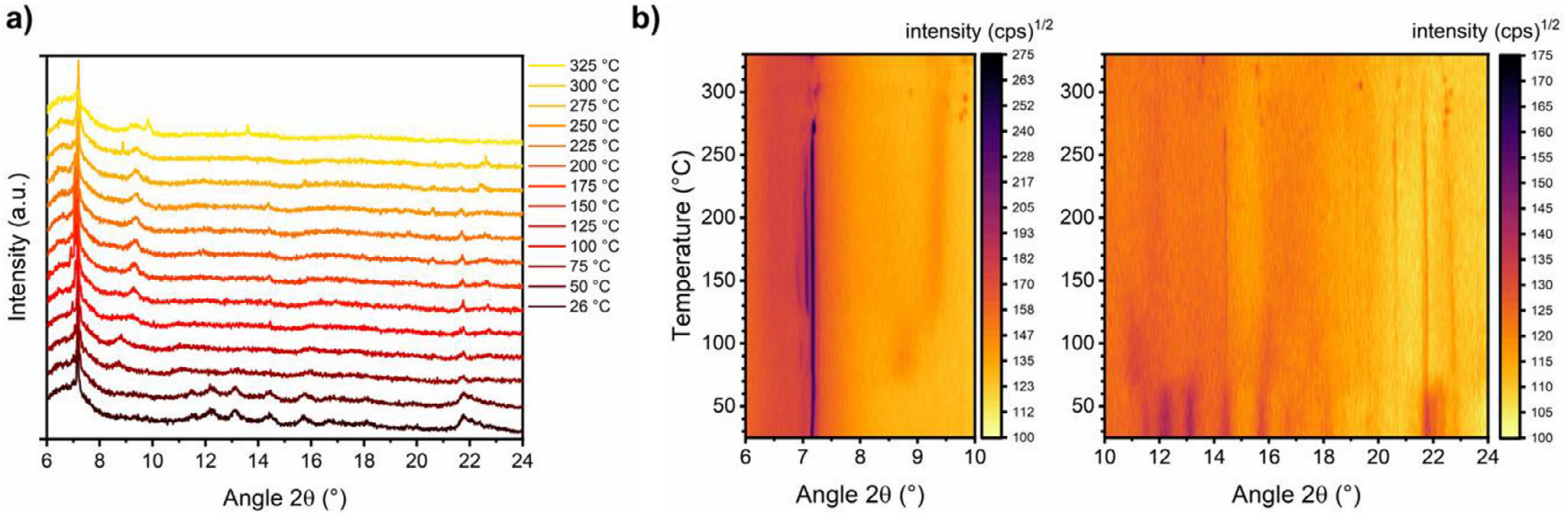
a) In situ VT‐PXRD (Cu *K_α_* radiation) in a staggered plot until 330 °C, showing no drastic changes in the patterns over a long 2*θ* range. b) Contour plots of TUB41 from 25–330 °C using square‐root scaling of the intensity, allowing us to better follow changes in crystallinity and phase transformations. The dark areas correlate 2*θ* values to observed high intensity of reflections.

**Figure 5:**
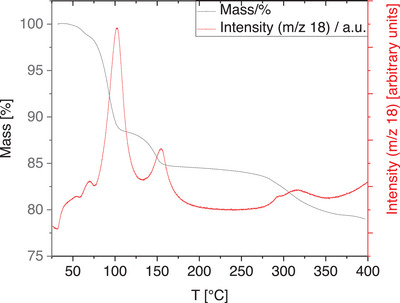
MS‐TGA measurements in synthetic air for TUB41.

To gain insight into the stability of the material after several years of cycling, we performed in situ variable temperature powder X‐ray diffraction (VT‐PXRD) on 2 year old crystals, which were repeatedly used in CO_2_ and water vapor adsorption experiments (∼20 cycles in total) and heated up to 330 °C. As can be seen in Figure 4a, the PXRD pattern remains very similar to the original phase until 330 °C, although some minor phase transformations can be observed. The phase transformation and thermal stability of the compound is better observed in contour plots of TUB41 in Figure 4b, with the dark areas showing crystalline, consistent Bragg reflections with varying intensity continuing until 330 °C. The most interesting reflex is observed at 2*θ* = 7.2°, which splits into two peaks above 120 °C and reverts to one peak above 250 °C, demonstrating the strongest phase transformation. Between 14° and 25°, many smaller but observable transformations occur. At 2*θ* = 14.4°, 20.5°, 21.8°, and 22.4°, reflections are sharpening from temperatures above 80 °C, demonstrating higher crystallinity of the sample until 330 °C (see Figure 4b). We previously summarized the conformational changes in flexible rod‐shaped SBUs observed in phosphonate MOFs.^[^ ^16^ ^]^ The observed phase transitions might be a result of the reversible conformational changes in the Cu–O–P–O–Cu–O–P–O rings up to 250 °C. Due to the observed condensation above 250 °C, the crystal data becomes more stable with the formation of new peaks and retains the major patterns observed at low temperatures (see Figures 2, 4 and 5). As depicted in Figure 1b, the rod‐shaped SBU of TUB41 has repeating hydrogen bonds between the mono‐deprotonated phosphonate‐based hydrogen atom and Cu(II)‐coordinated phosphonate oxygen atoms. The thermal flexibility of TUB41 might be originating from the breaking and reforming of the hydrogen bonds leading to multiple SBU conformations and phase transitions at different temperatures.

## MS‐Coupled Thermogravimetric Analysis

We performed MS‐coupled thermogravimetric analysis (MS‐TGA) to understand the thermal behavior of TUB41 (see Supporting Information for instrumental details). As depicted in Figure 5, the MS‐TGA results show two distinct steps of mass loss, occurring at around 100 °C (11.5 %) and around 150 °C (3.5%), which is identified as loss of water. After ca. 250 °C, decomposition is observed, which is accompanied by a loss of water, probably due to the condensation of phosphonic acid groups.^[^ ^36^, ^43^ ^]^ This transition is also confirmed by the in situ temperature variable PXRD showing peaks after 200 °C, and more intensely after keeping the TUB41 crystals at 250 and 300 °C for a longer period of time (see Figure 2b). At 400 °C, still more than 75 mass% of the sample remains, indicating appreciable thermal stability of the organic linker molecules. However, the structure has potentially changed to a less crystalline phase after condensation of the phosphonic acids, according to the PXRD results above 250 °C (see Figures 2b and 4).

## Adsorption Studies

To characterize the surface area and porosity of TUB41, nitrogen and argon adsorption isotherms were measured at 77 K. As seen in Figure S4, they show very low adsorption and yield a BET surface area of only ca. 4 m^2^ g^─1^ and a total pore volume of 0.04 cm^3^ g^−1^, consistent with low porosity. In addition, nitrogen adsorption isotherms measured at 298 and 313 K (see Figure S4) also show very low adsorption, indicating that TUB41's pores continue to exclude gases with larger kinetic diameters even at elevated temperatures.

**Figure 6:**
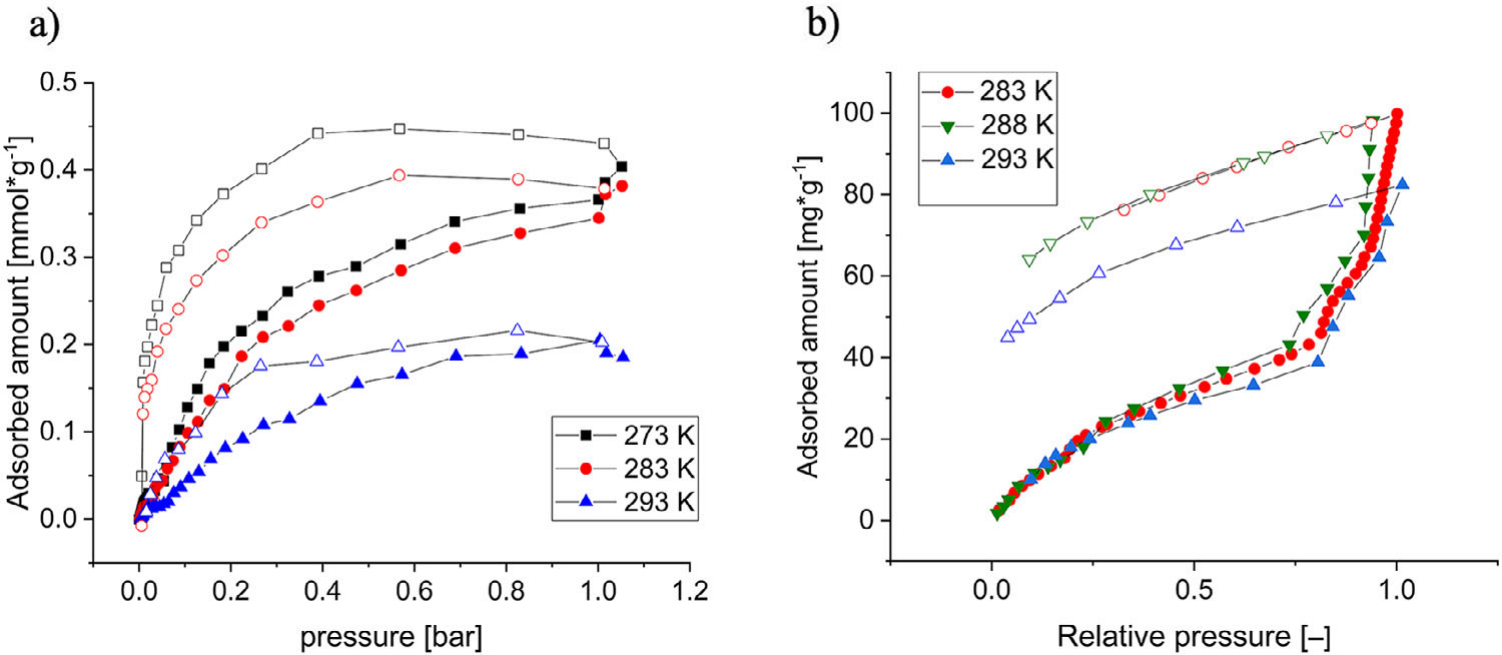
a) CO_2_ adsorption isotherms at different temperatures (filled symbols–adsorption, empty symbols–desorption). b) Water vapor adsorption isotherms at different temperatures (filled symbols–adsorption, empty symbols–desorption).

In contrast, the CO_2_ adsorption isotherms (Figure 6a) show a higher adsorption and distinct hysteresis over the whole pressure range, indicating an attractive interaction between the adsorbent and adsorbate. The hysteresis looks closest to type H4,^[^ ^44^, ^45^ ^]^ further strengthening the hypothesis of small, narrow pores with a polar internal surface. BET calculations based on the CO_2_ adsorption isotherm at 273 K yield a surface area of 60 m^2^ g^−1^ and a total pore volume of 0.019 cm^3^ g^−1^, consistent with low porosity. At low loadings (0.01 mmol g^−1^ CO_2_), the enthalpy of adsorption was estimated to be −41 kJ mol^−1^, indicative of a strong attractive interaction between CO_2_ and the framework. (The details for calculating the enthalpy of adsorption, including model fitting and application of the Clausius–Clapeyron equation, are provided in the Supporting Information). This suggests the presence of high‐affinity binding regions within the pores, making TUB41 suitable for selective CO_2_ capture in low‐pressure environments. For comparison, TUB41's enthalpy of adsorption is comparable to that of CALF‐20, which is −39 kJ mol^−1^.

Water vapor isotherms measured at 283, 288, and 293 K exhibit a gradual uptake in the low‐pressure region, followed by a sharp rise at ∼0.8 relative pressure, a step at ∼0.9, and another sharp rise at higher relative pressures (Figure 6b). This behavior matches a combination of type IV and type II isotherms at lower (up to ∼0.8) and higher relative pressure, respectively. For water vapor sorption, type IV and type II isotherms are indicative of a hydrophilic material.^[^ ^46^ ^]^ Importantly, the water uptake until ∼0.8 relative pressure of 0.04 g g^−1^ matches very well with the pore volume of 0.04 cm^3^ g^−1^ from N_2_ gas sorption at 77 K (vide supra), thereby supporting the micropore filling with (“liquid”) water (at a density of ∼1 g cm^−3^) until this relative pressure. Subsequently, at relative pressures between 0.7 and 0.8, inter‐particle condensation in meso‐ and macro‐pores begins, leading to a strong increase in the amount of adsorbed water in line with the type II branch starting at this relative pressure. The desorption measurements show a pronounced hysteresis, closest to type H2 behavior.^[^ ^43^, ^45^ ^]^ The incomplete desorption observed at all temperatures is indicative of strong interactions between the water molecules and polar groups within the pores. Since the same sample was used for all measurements, it was found that reactivation at 80 °C under vacuum for 2 h was sufficient to empty the pores. The total water uptake exceeds that of CO_2_ and is much higher than that of nitrogen, underscoring the selectivity of the pores for molecules with sufficiently small kinetic diameters at elevated temperatures.

The enthalpy of adsorption of water vapor was estimated using the same procedure as for CO_2_, focusing on the low‐pressure region prior to the onset of condensation. At a loading of 0.7 mmol g^−1^ (corresponding to a pressure of 2 × 10^−3^ bar), the calculated enthalpy of adsorption is −38 kJ mol^−1^. This higher loading, relative to the CO_2_ case, was selected due to the lower accuracy in the low‐pressure region of the water sorption and the available measurement points (Figure S2). The enthalpy of adsorption increases with increasing pressure (Figure S3), probably due to the formation of hydrogen bonds between adsorbed water molecules.

TUB41 exhibits a higher mass of water vapor adsorption (0.04 g g^−1^) compared to CO_2_ adsorption (0.017 g g^−1^), which might be due to the smaller kinetic diameter of water molecules (2.65 Å) compared to CO_2_ (3.3 Å) in the gaseous phase. Another reason for the higher water adsorption might be due to the condensation of water molecules between the TUB41 crystals, which usually happens at higher relative pressures. It should be noted that other MOFs, such as CALF‐20, which capture CO_2_ via physisorption, also adsorb a higher amount of H_2_O compared to CO_2_.^[^ ^15^ ^]^ As discussed below, our MD simulations suggest that this phenomenon may be due to the formation of hydrogen bonds between water molecules, leading to more densely packed water molecules in the pores. On the other hand, gases like CO_2_ interact weakly with each other via noncovalent interactions.

The total number of MOFs that are more selective for CO_2_ in the presence of water vapor is very limited in the literature, as MOFs usually have higher affinity for water vapor due to the presence of hydrophilic moieties.^[^ ^15^, ^22^ ^]^ Although the amount of adsorbed CO_2_ is limited in TUB41 due to its small surface area, it still has a unique place among MOFs with its narrow and selective pores limiting competition with gases having larger kinetic diameters, e. g., nitrogen (3.64 Å) under cryogenic conditions and methane (3.8 Å). Moreover, the naphthalene moieties impart a high hydrophobicity to the pores.

Altogether, the adsorption measurements confirm the existence of very small pores, which are not accessible to nitrogen (under both cryogenic and higher measurement temperatures) and argon (under the cryogenic measurement temperatures) due to their larger kinetic diameter (see Figure S4). Argon has a slightly larger kinetic diameter of 3.4 Å compared to CO_2_ which has a kinetic diameter of 3.3 Å.^[^ ^47^ ^]^ Therefore, TUB41 excludes gases with kinetic diameters exceeding approximately 3.3–3.4 Å.

## Molecular Dynamics Studies

We performed molecular dynamics (MD) simulations on several TUB41 systems (12 × 4 × 4 supercells) containing varying concentrations of H_2_O and CO_2_, employing the flexible UFF4MOF force field for TUB41, rigid TIP4P model for water, and TraPPE model for CO_2_. Specifically, four systems with different H_2_O concentrations (containing 10, 30, 100, and 300 water molecules) and three systems with different CO_2_ concentrations (containing 10, 30, and 100 CO_2_ molecules) were constructed to investigate the distribution, adsorption, and diffusion properties of H_2_O and CO_2_ in TUB41. The full simulation details are provided in the Supporting Information.

**Figure 7:**
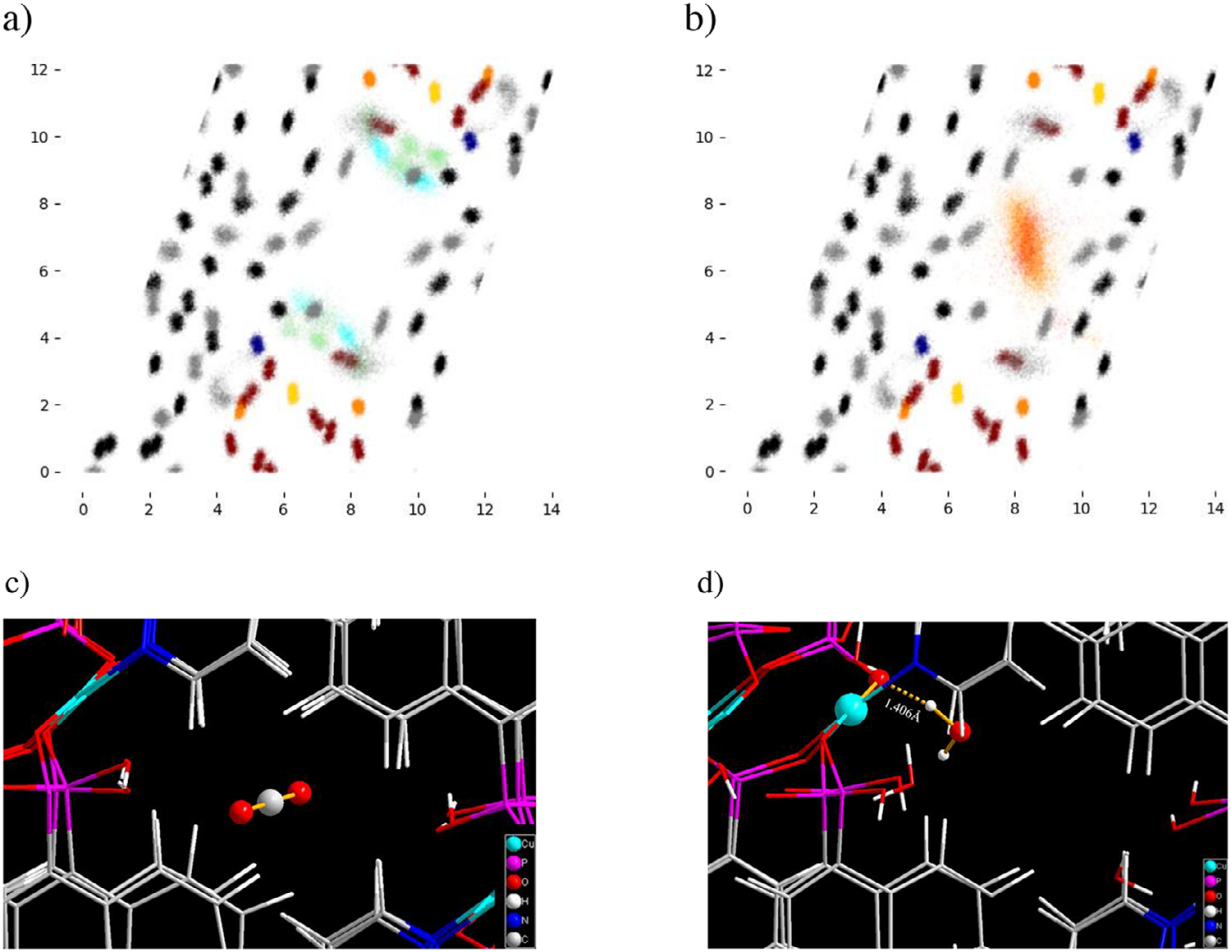
Atomic trajectory overlay maps of a TUB41 unit cell containing a) 30 H_2_O molecules and b) 30 CO_2_ molecules. The maps are projections onto a plane that is perpendicular to the channel direction. Cyan and green represent the oxygen and hydrogen atoms of H_2_O, respectively; red and orange represent the carbon and oxygen atoms of CO_2_, respectively; gold, dark blue, dark orange, dark red, black, and gray represent copper, nitrogen, phosphorus, oxygen, carbon, and hydrogen atoms, respectively, in the MOF framework. The *x* and *y* axes of the maps represent position, expressed in Ångströms (Å). A representative snapshot from the MD trajectory showing c) a typical position of a CO_2_ molecule within a pore, and d) a typical hydrogen bond at the edge of a pore, formed between a H_2_O molecule and an oxygen atom in the MOF's SBU.

To visualize the distribution of CO_2_ and H_2_O molecules within the pores of TUB41, atomic trajectory overlay maps were generated for each system. Specifically, the positions of all atoms in the supercell were saved at every 1000 frames of a trajectory. Then, for each unit cell, the positions of all atoms were re‐calculated relative to a common reference point in the unit cell, and these relative positions were projected onto a two‐dimensional plane perpendicular to a given axis. Finally, the atomic trajectory overlay map is generated by superimposing the projections for each unit cell in the supercell onto each other. An example of a trajectory overlay map for the system containing 30 H_2_O and 30 CO_2_ molecules (projected onto a plane perpendicular to the *x*‐axis, i.e., channel direction) is shown in Figure 7. Atomic trajectory overlay maps for the remaining systems and projections are provided in the Supporting Information.

After analyzing the trajectory overlay maps for all systems and projections, several interesting conclusions can be drawn: the orientations and positions of the H_2_O and CO_2_ molecules are significantly constrained by the narrow channels, resulting in ordered arrangements. The H_2_O molecules preferentially localize near the SBUs, whereas the CO_2_ molecules predominantly occupy the centers of the channels (as seen in Figure 7). This distribution pattern is observed across all concentrations, suggesting distinct adsorption mechanisms for H_2_O and CO_2_ in the TUB41 framework.

To further investigate the distribution and interactions of the CO_2_ and H_2_O molecules with the TUB41 framework, radial distribution functions (RDFs) for select atomic pairs were calculated. The RDF for a pair of atoms *a* and *b*, *g*_ab_(*r*), is defined as^[^ ^40^, ^41^ ^]^

(1) $$g_{\mathrm{ab}}\left(r\right)={\left(N_{a}N_{b}\right)}^{-1}\sum_{i=1}^{N_{a}}\sum_{i=1}^{N_{b}}\langle \delta \left(\left|r_{i}-r_{j}\right|-r\right)\rangle$$

where *r* is the distance from atom *a*, *N*_a_ and *N*_b_ are the number of *a* and *b* atoms, respectively, ***r**_i_* and ***r**_j_* are the position vectors of particles *i* and *j*, respectively, and *δ* is the Dirac delta function.

**Figure 8:**
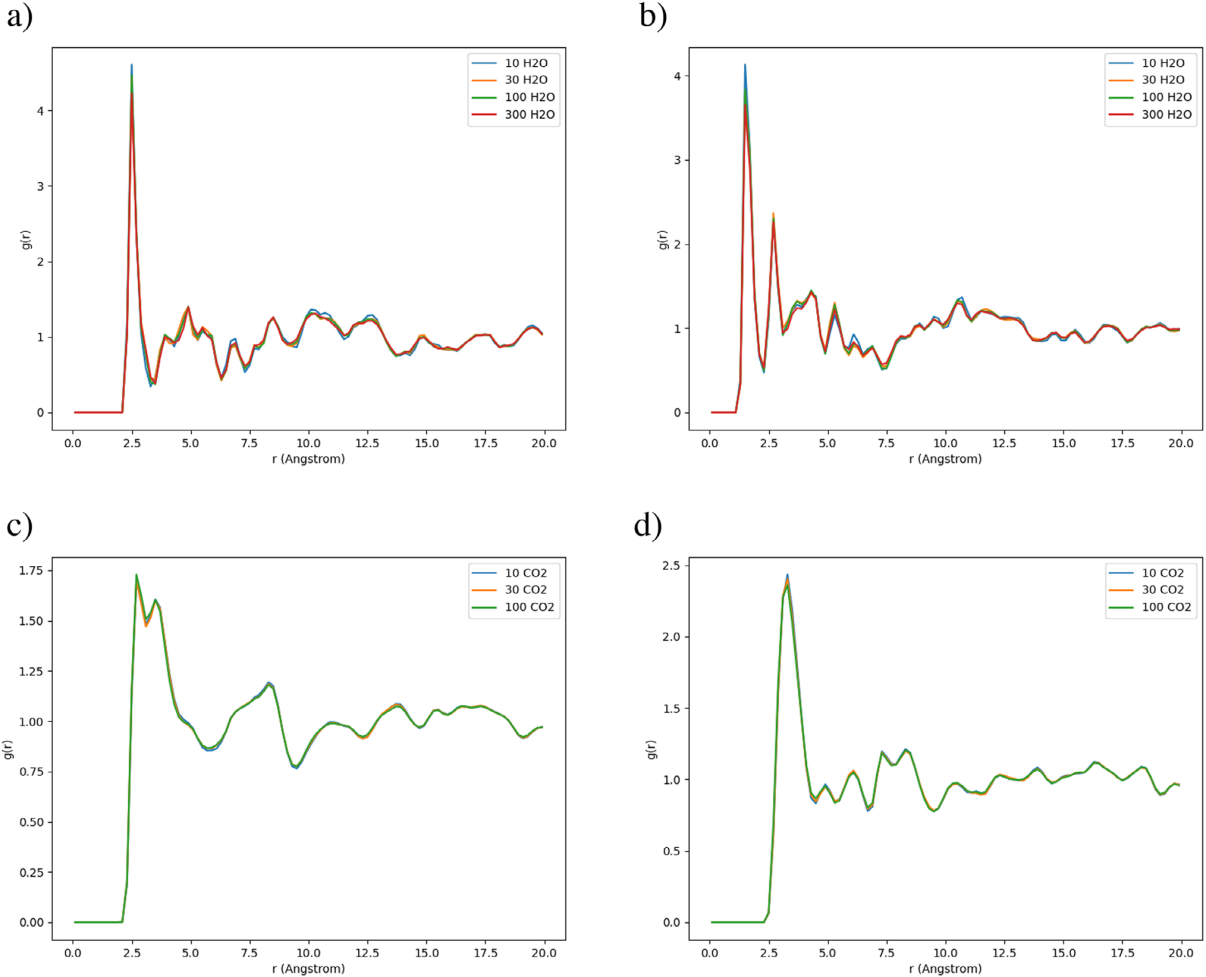
RDFs between atoms in CO_2_/ H_2_O and atoms in the TUB41 framework for all systems. a) O (H_2_O)–O (framework). b) H (H_2_O)–O (framework). c) O (CO_2_)–H (framework). d) C (CO_2_)–H (framework).

Figure 8 presents RDFs between atoms in CO_2_/H_2_O and atoms in the TUB41 framework for all systems studied. The O (H_2_O)–O (framework) RDF possesses a distinct peak at around 2.5 Å, indicating the presence of strong hydrogen bonding (see Figure 8a). In contrast, no significant peaks below 3.5 Å were observed between O (H_2_O) atoms and other framework atoms (see Figure S29). The H (H_2_O)–O (framework) RDF possesses two distinct peaks at around 1.5 and 2.7 Å, further supporting the presence of hydrogen bonding (see Figure 8b). Figure 8c shows two close peaks between 2.5–3.5 Å due to significant nonbonding interactions between the two O (CO_2_) atoms and multiple H (framework) atoms; similarly, Figure 8d shows a single peak around 3 Å due to significant nonbonding interactions between the C (CO_2_) atom and multiple H (framework) atoms. This explains the ordered arrangement of CO_2_ molecules in the channel centers observed in Figure 7b. (For more RDF plots, the reader is referred to Figure S29 of the Supplementary Information.)

**Figure 9:**
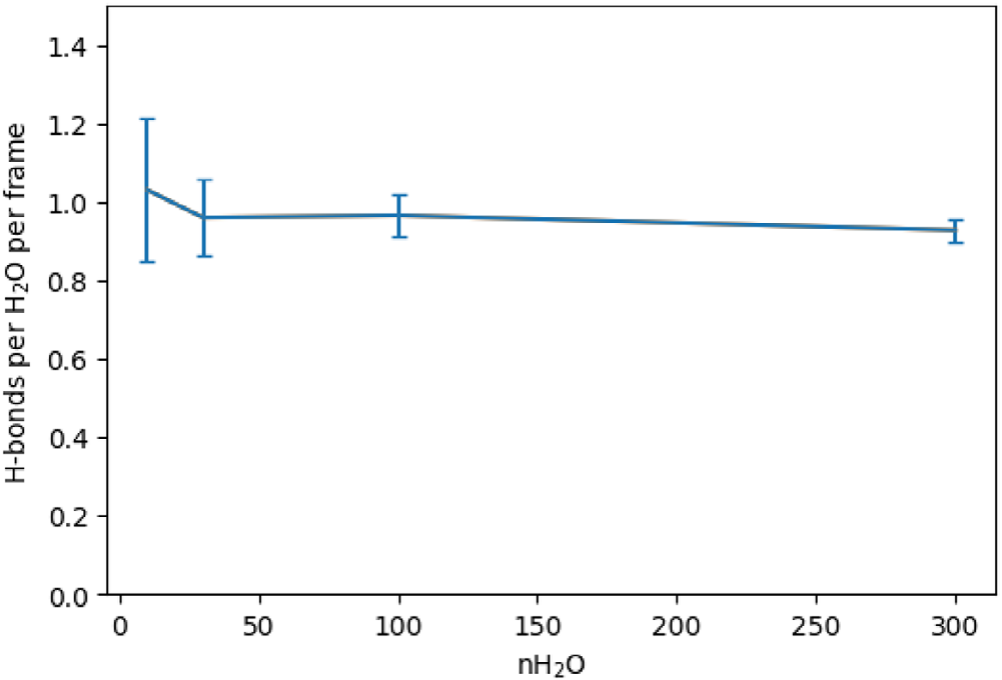
Average number of hydrogen bonds between a H_2_O molecule and the MOF framework for the water‐containing systems, normalized by both the number of trajectory frames and the number of H_2_O molecules.

We next quantified the average number of hydrogen bonds between a H_2_O molecule and the MOF framework for the water‐containing systems.^[^ ^42^ ^]^ Hydrogen bonds were assigned according to the following criteria: (1) donor–acceptor distance less than 3.0 Å, and (2) donor–hydrogen–acceptor angle greater than 150°. The results are shown in Figure 9. Across all investigated systems, each water molecule forms, on average, about one hydrogen bond with the MOF framework, consistent with the experimental hydration results. Based on the RDF results, this bond is a strong hydrogen bond. Additionally, no hydrogen bonds were identified between CO_2_ molecules and the MOF framework (result not shown). We also analyzed the average number of hydrogen bonds between H_2_O molecules for the water‐containing systems. For the 30‐ and 100‐water systems, this number is essentially zero; however, for the 300‐water system, there are ∼0.065 bonds, a small but significant number (see Figure S30). This is because, at higher concentrations, the binding sites of the framework become saturated, causing the additional water molecules to reside in the center of the channel where they have more opportunity to hydrogen bond with each other. This phenomenon is further supported by the atomic trajectory overlay map of the 300‐water system (see Figure S28).

**Figure 10:**
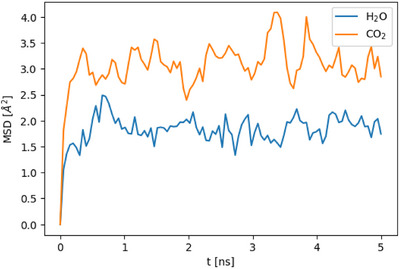
MSDs of H_2_O and CO_2_ in the TUB41 systems containing 30 H_2_O and 30 CO_2_ molecules, respectively.

The mean‐squared displacements (MSDs) of H_2_O and CO_2_ were computed according to:^[^ ^43^, ^44^ ^]^

(2) $$MSD\left(t\right)=\frac{1}{N}\sum_{i=1}^{N}{\left|r_{i}-r_{i}\left(t\right)\right|}^{2}$$

where *N* is the number of particles averaged over and *r_i_* is the position of particle *i*. They were calculated based on 5 ns NVT trajectories, started from the final frames of the previous production runs. The MSD results for the systems containing 30 H_2_O and 30 CO_2_ molecules are shown in Figure 10. Both the H_2_O and CO_2_ MSDs exhibit similar trends. Initially, the MSDs increase steadily up to about 0.3 ns. Beyond 0.3 ns, both MSDs cease to grow further and instead fluctuate around equilibrium values. The equilibrium MSD value is approximately 2.0 Å^2^ for H_2_O and 3.2 Å^2^ for CO_2_. These values are exceptionally small, indicating that both H_2_O and CO_2_ molecules are tightly confined in all three spatial dimensions near their equilibrium positions within the pores. This confinement arises from a combination of strong guest–host interactions and the ultramicroporous character of TUB41. For comparison, in another MOF currently under investigation, the MSDs of H_2_O and CO_2_ reach up to 1000 Å^2^ within 5 ns. Given both the low MSD values observed in TUB41 and the small difference between them, it is not possible to draw meaningful conclusions about any preferential kinetic behavior between the two gases in this system.

In addition to MD simulations, we employed the random insertion algorithm in LAMMPS to estimate the maximum adsorption capacities of CO_2_ and H_2_O in the TUB41 supercell. For each gas, 1000 insertion attempts were made, with up to 10 000 placement attempts per molecule. If a suitable position is not found after 10 000 attempts, the algorithm proceeds to the next molecule. A minimum initial distance of 1.6 Å between all atoms was enforced to ensure stability, as values below this threshold resulted in unstable simulations. Parallel simulations were conducted using three different random number seeds. The number of CO_2_ molecules successfully inserted into the TUB41 supercell was 254, 244, and 242, with an average of 247, while for H_2_O, the counts were 762, 766, and 766, averaging 765. Thus, the calculated maximum adsorption capacities are 0.084 g g^−1^ for H_2_O and 0.066 g g^−1^ for CO_2_, which are consistent with the experimental observation that TUB41 possesses a higher mass of water vapor adsorption (0.04 g g^−1^) compared to CO_2_ adsorption (0.017 g g^−1^).

Overall, the MD simulation results suggest that the pores of TUB41, which are lined predominantly with hydrophobic groups and only at select sites with hydrophilic phosphonate oxygen atoms from the SBUs, promote spatial separation between H_2_O and CO_2_, potentially enabling coadsorption while minimizing competitive interactions.

## Optical Properties

Copper phosphonate MOFs, such as TUB1, TUB40, and TUB75 (sister compounds of TUB41), and other phosphonate MOFs are known for their semiconducting properties.^[^ ^33^, ^37^, ^38^, ^48^ ^]^ Thus, we measured the diffuse reflectance spectrum of TUB41 to estimate its optical band gap. Based on the Tauc plots generated from the diffuse reflectance spectra (see Figures S21a and S21b), we extracted an indirect band gap of 2.7 eV and direct band gap of 3.0 eV (see Supporting Information for experimental details).^[^ ^49^, ^50^ ^]^ The band centered at around 1.7 eV is likely due to a localized 3d^9^←3d^9^ transition of the Cu(II) centers (^2^E←^2^T_2_ in an approximately tetrahedral field). TUB41 has a very similar SBU compared to our previously reported MOF TUB1. The SBUs in both TUB1 and TUB41 have the same order of vertex‐connected [Cu–O–P–O–Cu–O–P–O] eight‐membered rings resulting in a rod‐shaped SBU (see Figure 1b).^[^ ^48^ ^]^ The major difference between the SBUs of TUB41 and TUB1 is the first copper atom (Cu1) in TUB1, which is coordinated in a square‐planar fashion, while the second copper atom (Cu2) is coordinated in a square‐pyramidal fashion. In TUB41, all copper atoms show a square–pyramidal coordination sphere. Similar to TUB41, our previously published MOF TUB1 possesses an indirect band gap of 2.4 eV and a direct band gap of 2.7 eV, which was also confirmed by DOS calculations.^[^ ^48^ ^]^ The slightly narrower band gap observed in TUB1 might be due to the presence of square planar coordinated Cu(II) ions as indicated by the calculations in the previous work. The indirect and direct band gaps of TUB41 are within the visible range, highlighting the potential of TUB41 in photocatalysis applications.

## Conclusions

TUB41 stands out among MOFs for its narrow, selective pores, which effectively exclude gases with kinetic diameters larger than 3.3 Å, and for its remarkable stability, maintaining structural integrity over a two‐year period. It remains stable for over a month at pH 2.5 and for at least 2 h across a broad pH range from 1 to 11. The crystals also retain their stability after repeated adsorption experiments and activation at 80 °C under vacuum. Compared to conventional MOFs, which are often hydrolyzed by water vapor, TUB41 shows superior chemical stability, particularly in acidic and basic environments within the pH 1–11 range. This exceptional stability enhances its suitability for industrial applications, especially in membrane‐based coseparation of CO_2_ and H_2_O from other gases. In contrast, typical MOFs with metal‐carboxylate SBUs, such as UiO‐66, have relatively short shelf lives of approximately 2 months under ambient conditions.

A major challenge in CO_2_ capture under humid conditions is the typically stronger affinity of MOFs for water, largely due to the presence of hydrophilic metal‐binding groups. In TUB41, the presence of naphthalene moieties lining the pores imparts a predominantly hydrophobic character to the pores, creating a more favorable environment for CO_2_ adsorption. The hydrophilic oxygen atoms of the phosphonate groups cover only a small fraction of the inner surface area (Figure 1a,c), which is sufficient to enable water adsorption while preserving the overall hydrophobic character of the pores. The slit‐shaped channels along the *a* axis, with a cross‐section of approximately 4 × 2 Å^2^, provide a confined space well‐suited for the selective adsorption of gases with small kinetic diameters. Additionally, the rod‐shaped SBU imparts TUB41 with reversible phase transitions observable at different temperatures. In situ temperature‐variable PXRD combined with MS‐TGA points to the condensation of phosphonate groups, leading to the release of additional water molecules at elevated temperatures.

The MD simulations reveal distinct adsorption mechanisms for H_2_O and CO_2_ within TUB41. H_2_O preferentially localizes near the SBUs through strong hydrogen‐bonding interactions, while CO_2_ predominantly occupies the central regions of the channels, driven by Coulombic and van der Waals forces. The RDFs and hydrogen‐bond analysis confirm the specificity and strength of these interactions. The MSD results further demonstrate that both H_2_O and CO_2_ are spatially constrained within the pores, highlighting their strong affinity for the MOF framework. Despite the higher loading capacity of H_2_O and its strong hydrogen‐bonding interactions, which may impact CO_2_ uptake, the largely hydrophobic nature of TUB41's pores and the spatial separation between CO_2_ (in the center of the channels) and H_2_O (near the SBUs) suggest that competitive adsorption could be partially mitigated. These characteristics enable the selective adsorption of CO_2_ in humid environments, highlighting TUB41's potential for gas separation applications.

In summary, TUB41 represents a significant step forward in the design of robust MOFs, combining exceptional long‐term thermal and chemical stability with selective gas adsorption and a visible‐range band gap. Its unique combination of properties–including over 2 years of structural stability, resistance to harsh chemical environments, and molecular‐level selectivity for CO_2_ and water vapor–positions it as a promising candidate for applications such as photocatalytic water splitting, CO_2_ reduction, and industrial gas separation. The development of phosphonate‐based MOFs like TUB41, featuring insoluble SBUs and resilience in concentrated acids and bases, paves the way for the next generation of MOFs capable of operating in demanding aqueous and chemical environments.

## Supporting Information

The Supporting Information contains details of the synthesis, gas and water adsorption, MD simulations, and optical spectroscopy.

## Author Contributions

R.O. performed the gas sorption studies and optical measurements, and wrote the corresponding sections. M.F. resynthesized TUB41, developed a new method for the linker synthesis, and performed the stability experiments and took SEM pictures. Y.Z. set up and performed the MD simulations, generated the corresponding figures, and wrote the initial drafts of the computational parts of the manuscript. A.S. performed the N_2_ and Ar adsorption measurements, A.K. performed the in situ temperature variable PXRD measurements and wrote the corresponding section, M.S. supervised the optical measurements, wrote the corresponding section, and edited the manuscript. C.J. supervised and interpreted the gas adsorption work, created the crystallographic figures, and edited the manuscript. G.H. supervised the work of Y.Z., provided critical feedback on several experimental sections of the manuscript, revised the computational parts of the manuscript, and edited the entire manuscript. G.Y. created the hypothesis, supervised the work of M.F. and R.O., and wrote the majority of the abstract, introduction, conclusion sections, and contributed experimental sections of the manuscript.

## Conflict of Interests

The authors declare no conflict of interest.

## Supporting information



Supporting Information

## Data Availability

The data that support the findings of this study are available in the Supporting Information of this article.
