# Supplementary material for: Stable Ultramicroporous Metal–Organic Framework with Hydrophilic and Hydrophobic Domains for Selective Gas Adsorption
Source: Angew Chem Int Ed Engl. 2025 Aug 23;64(40):e202513788. doi: 10.1002/anie.202513788 (PMC12462748; doi:10.1002/anie.202513788)
Supplement: Supplementary file 1 — Supporting Information [file ANIE-64-e202513788-s001.docx]

**Stable Ultramicroporous Metal-Organic Framework with Hydrophilic and Hydrophobic Domains for Selective Gas Adsorption**

Robert Oestreich,^1#^ Marcus N. A. Fetzer,^1#^ Yifei Zhang^6^, Andreas Schreiber,^3^ Alexander Knebel,^4,5^ Markus Suta,^2^ Christoph Janiak^1^*, Gabriel Hanna^6^*, and Gündoğ Yücesan^1^*

1. Institute for Inorganic and Structural Chemistry, Heinrich Heine Universität Düsseldorf, Universitätsstr. 1, D-40225 Düsseldorf, Germany.

2. Inorganic Photoactive Materials, Institute for Inorganic and Structural Chemistry, Heinrich Heine University Düsseldorf, Universitätsstr. 1, 40225 Düsseldorf, Germany

3. Microtrac Retsch GmbH, [Retsch-Allee 1-5, D-42781 Haan](https://www.google.com/maps/place/data=!4m2!3m1!1s0x47b8d23188c2fc73:0xecf2dc944c0cd77d?sa=X&ved=1t:8290&ictx=111), Germany

4. Otto Schott Institute of Materials Research, Center for Energy and Environmental Chemistry II, Friedrich Schiller University Jena, Lessingstraße 12-14, D-07743 Jena, Germany.

5. Center for Energy and Environmental Chemistry, Friedrich Schiller University Jena, Philosophenweg 7a, D-07743 Jena, Germany

6. Department of Chemistry, University of Alberta, Edmonton, Alberta, Canada

# These authors contributed equally to this work.

**Table of Contents**

1. General information
2. Synthesis
3. NMR spectra
4. Gas and water sorption
5. Optical spectroscopy
6. SEM images
7. In-situ variable temperature powder x-ray diffraction (VT-PXRD)
8. Molecular dynamics simulation
9. **General information**

Unless otherwise noted, all commercially available compounds were used as provided without further purification. Chemicals used in this study were purchased from Sigma Aldrich, Alfa Aesar, BLDpharm and Carl Roth. Solvents for chromatography were technical grade and distilled prior to use. Analytical thin-layer chromatography (TLC) was performed on Macherey-Nagel silica gel aluminium plates with F-254 indicator, visualized by irradiation with UV light. Column chromatography was performed using silica gel Merck 60 (particle size 0.063 – 0.2 mm). ^1^H-NMR, ^13^C-NMR and ^31^P-NMR were recorded on a Bruker Avance III 300 MHz NMR spectrometer in CDCl_3_ or DMSO-d_6_. Data are reported in the following order: chemical shift (δ) in ppm; multiplicities are indicated brs (broadened singlet), s (singlet), d (doublet), t (triplet), q (quartet), m (multiplet); coupling constants (J) are in Hertz (Hz). Powder X-ray diffraction (PXRD) was performed on a Rigaku Miniflex powder diffractometer in θ/2θ geometry with Cu-Kα radiation (1.54184 Å) and equipped with a rotating low-background silicon sample holder. For thermogravimetric analysis (TGA) of the pure MOF a Netzsch TG 209 F3 Tarsus was used and operated with synthetic air atmosphere and a heating rate of 10 K min^-1^ . TGA curves were baseline corrected with a blank run. Gaseous products were analyzed with a GAM 200 mass spectrometer from InProcess Instruments. Gas and vapor sorption measurements were done on the BELSorp-max II by MicrotracBEL Corporation. The sample was heated to 80°C under vacuum for two hours for activation before every measurement to clear pores of residue gas. Diffuse reflectance and emission spectra were measured on an Edinburgh FLS1000 luminescence spectrometer with 450 W Xe arc lamp, double grating monochromators (Czerny-Turner configuration, blazed at 400 nm in excitation and 500 nm in emission) and a thermoelectrically cooled (-20 °C) PMT980 (Hamamatsu) photomultiplier tube as detection unit. All spectra were acquired at room temperature. Diffuse reflectance was measured using an integrating sphere (diameter 120 mm) setup with the inner surface coated with BenFlect (reflectance >99 % between 350 nm and 2500 nm). All spectra were corrected for the grating efficiency, the lamp intensity, and the wavelength-dispersive sensitivity of the detection unit.

1. **Synthesis**

Synthesis of tetraethyl naphthalene-1,4-diylbis(phosphonate)

Under a nitrogen flow, 1,4-dibromonaphthalene (5,1 g, 17.8 mmol) was placed in a three-neck round-bottom flask equipped with a stirring bar. The flask was heated to 160 °C to melt the 1,4‑dibromonaphthalene. To the melted 1,4-dibromonaphthalene, NiBr_2_ (0.5 g, 2.2 mmol) was added. Finally, triethyl phosphite (7.8 g, 46.9 mmol) was added dropwise over a period of 7 h. The reaction was stirred at the same temperature for 24 h. The crude product, a dark brown to orange oil, was purified by column chromatography using a mixture of EtOAc and EtOH (9:1 v:v). The product was isolated as a colorless oil with a yield of 3.8 g (9.5 mmol, 53.3 %).

^1^H NMR (300 MHz, CDCl_3_) δ 8.63-8.59 (m, 2H), 8.29-8.22 (m, 2H), 7.68-7.64 (m, 2H), 4.27‑4.07 (m, 8H), 1.32 (t, *J* = 7.1 Hz, 12H); ^31^P{^1^H} NMR (121 MHz, CDCl_3_) δ 17.4 (s); ^13^C{^1^H} NMR (75 MHz, CDCl_3_) δ 132.9-132.4, 131.6 (d, *J* = 3.6 Hz), 129.2 (d, *J* = 3.5 Hz), 127.6, 127.3, 62.5 (d, *J* = 3.0 Hz), 16.3.

Synthesis of naphthalene-1,4-diphosphonic acid (1,4-NDPA-H_4_)

Tetraethyl naphthalene-1,4-diylbis(phosphonate) (3.8 g, 9.5 mmol) was mixed with 100 mL of 8 M hydrochloric acid and refluxed for 24 h. The white precipitate was filtered off and dried at 60 °C under reduced pressure to obtain 2.5 g (yield: 95 %) of pure acid.

^1^H NMR (300 MHz, DMSO-*d*_6_) δ 8.69-8.65 (m, 2H), 8.08-8.01 (m, 2H), 7.64 (dd, *J*=6.5, 3.4 Hz, 2H); ^31^P{^1^H} NMR (121 MHz, DMSO-*d*_6_) δ 11.0 (s);

^13^C{^1^H} NMR (75 MHz, DMSO-*d*_6_) δ 135.6 (d), 132.4 (d), 130.3‑129.9 (m), 127.8 (s), 126.4 (s).

1. **NMR spectra**

Tetraethyl naphthalene-1,4-diylbis(phosphonate)

^1^H NMR (300 MHz, CDCl_3_)

^13^C{^1^H} NMR (75 MHz, CDCl_3_)

^31^P{^1^H} NMR (121 MHz, CDCl_3_)

Naphthalene-1,4-diphosphonic acid (1,4-NDPA-H_4_)

^1^H NMR (300 MHz, DMSO-*d*_6_)

^13^C{^1^H} NMR (75 MHz, DMSO-*d*_6_)

^31^P{^1^H} NMR (121 MHz, DMSO-*d*_6_)

1. **Gas and water sorption**

Enthalpy of adsorption was estimated by measuring adsorption isotherms at three temperatures (273, 283, and 293 K), fitting the data to a Freundlich-Langmuir model (Figures S1 and S2), and applying the Clausius-Clapeyron equation:^39^

$\Delta H_{\mathrm{ads}}=R\ln\left( \frac{p_{2}}{p_{1}} \right)\frac{T_{1}T_{2}}{\left( T_{2}-T_{1} \right)}$ (1)

where *R* = 8.314 J ∙ K^-1^ ∙ mol^-1^ is the gas constant, *p*_1_ and *p*_2_ are two reference pressures, and *T*_1_ and *T*_2_ are the respective reference temperatures.

**Figure S1**: CO_2_ adsorption isotherms at different temperatures (on a semi-logarithmic scale), fitted to a Freundlich-Langmuir model.

**Figure S2:** Water adsorption isotherms at different temperatures (on a semi-logarithmic scale), fitted to a Freundlich-Langmuir model.


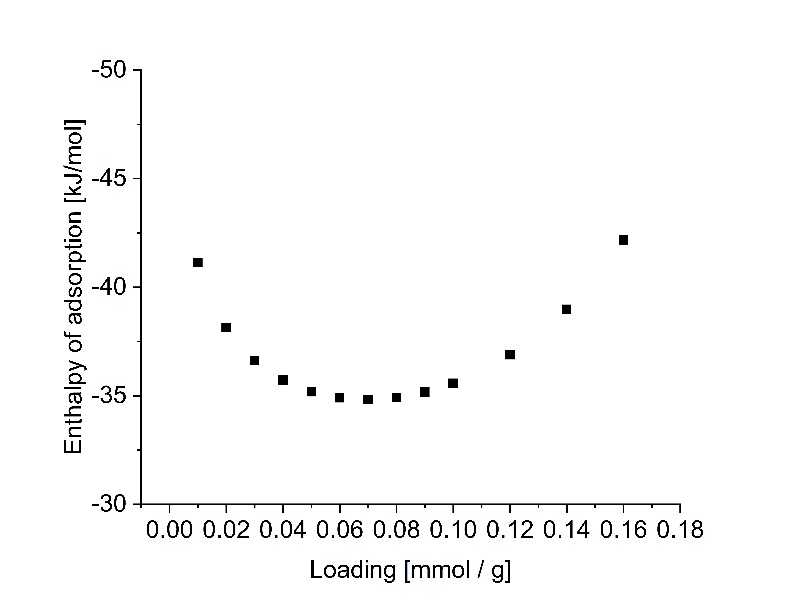

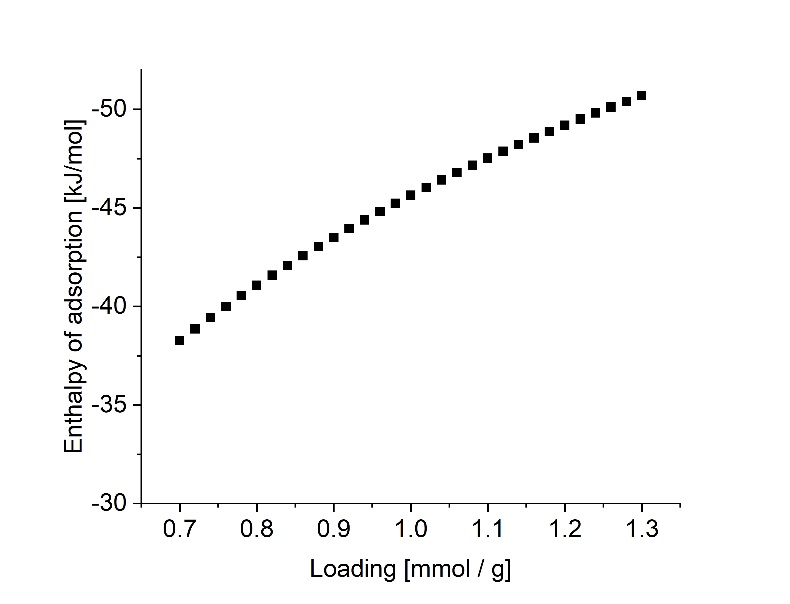


**Figure S3:** Enthalpy of adsorption against loading of CO_2_ (left) and H_2_O (right)


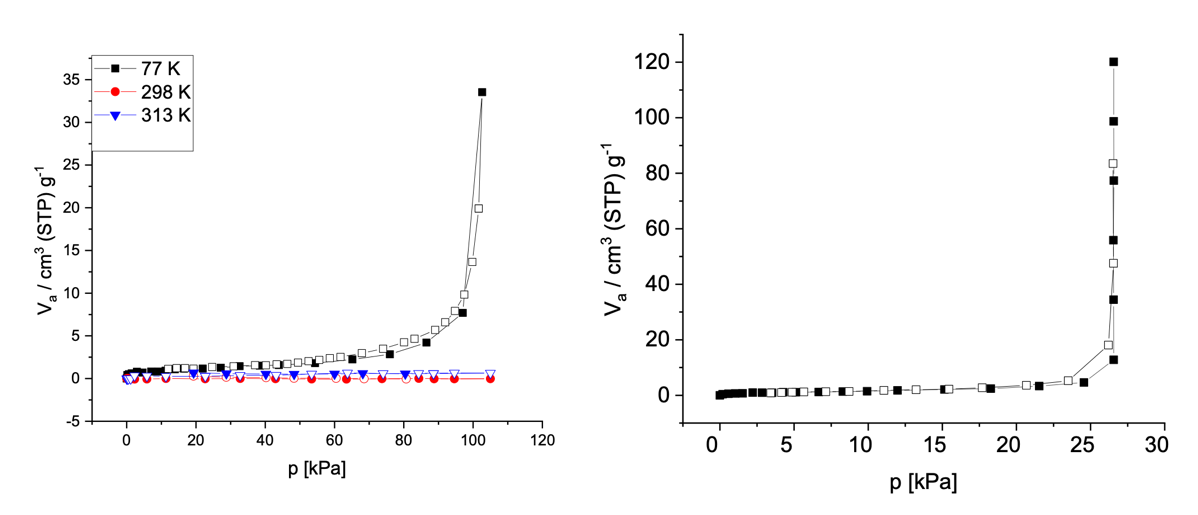


**Figure S4:** Nitrogen (left) and argon (right) adsorption isotherms for TUB41 (filled symbols – adsorption, empty symbols – desorption).

Below, we present the additional water and CO_2_ adsorption isotherms collected over a two-year period.

**

**Figure S5:** Water adsorption isotherm 1 at 283 K, before condensation

**Figure S6:** Water adsorption isotherm 2 at 283 K, before condensation

**Figure S7:** Water adsorption isotherm 1 at 288 K, before condensation

**Figure S8:** Water adsorption isotherm 2 at 288 K, before condensation

**Figure S9:** Water adsorption isotherm 1 at 293 K, before condensation

**Figure S10:** Water adsorption isotherm 2 at 293 K, before condensation

**Figure S11:** Water adsorption isotherm 1 at 298 K, before condensation

**Figure S12:** Water adsorption isotherm 2 at 298 K, before condensation

**Figure S13:** Water adsorption isotherm 1 at 303 K, before condensation

**Figure S14:** Water adsorption isotherm 2 at 303 K, before condensation

**Figure S15:** Water adsorption isotherm at 313 K, before condensation

**Figure S16:** Carbon dioxide adsorption isotherm at 313 K

**Figure S17:** Carbon dioxide adsorption isotherm at 313 K


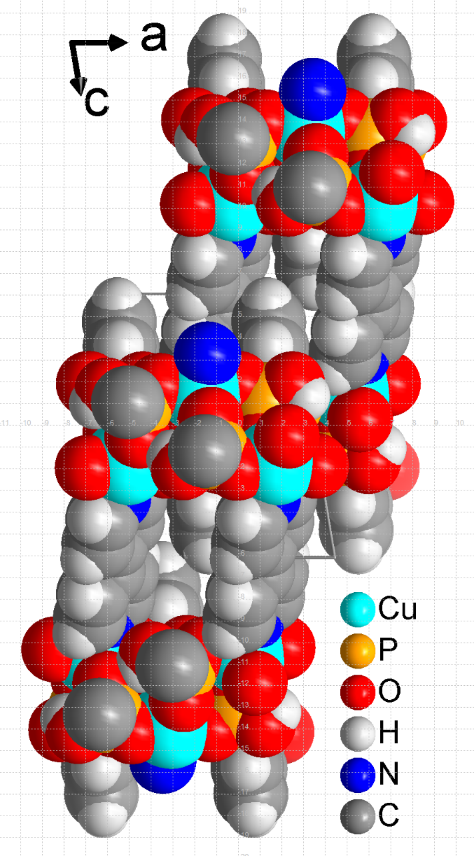

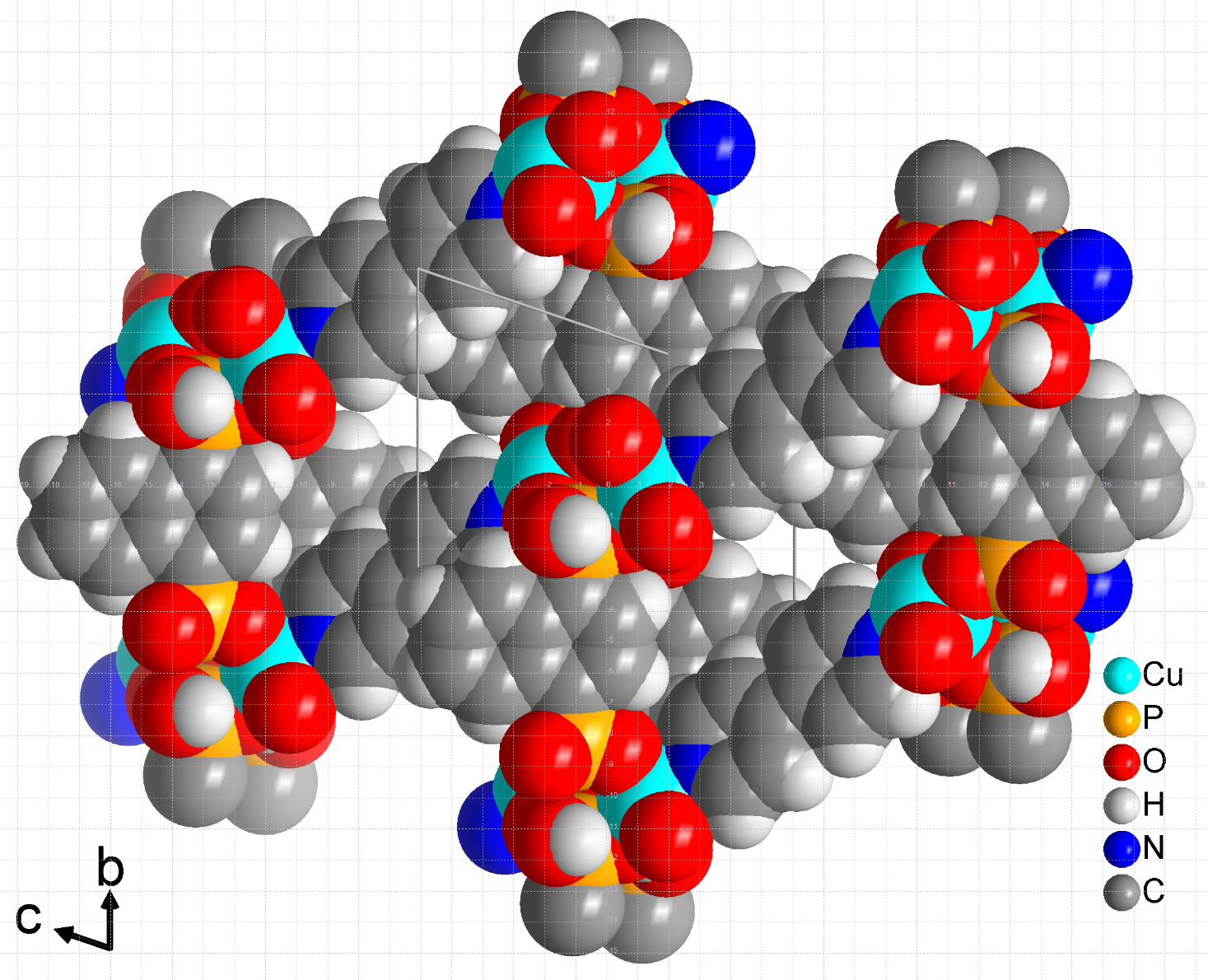


**Figure S18:** Section of the packing diagram in space-filling mode in two viewing directions to show the pore structure in the 3D network of [Cu(4,4’‐bpy)_0.5_(1,4‐NDPAH_2_)]. The edges of the grid-squares are 1 Å in length.


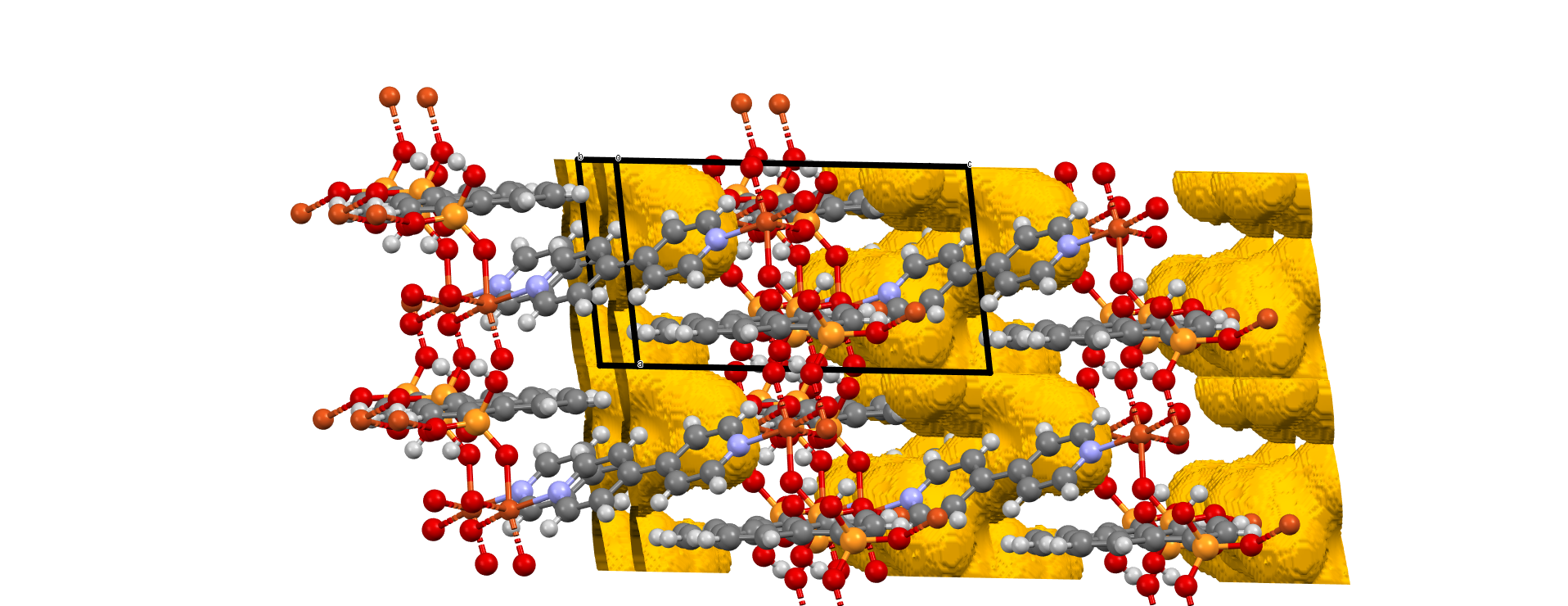


a

c


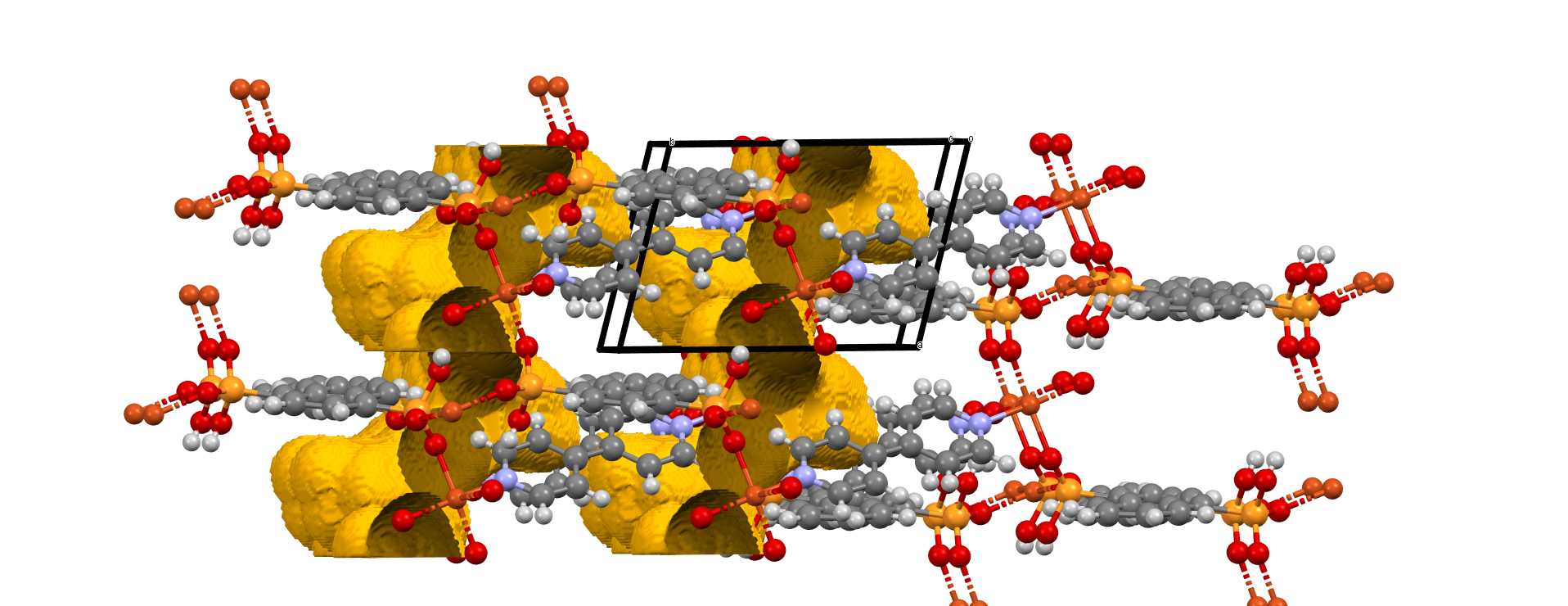


b

a

**Figure S19:** Voids in the MOF [Cu(4,4’‐bpy)_0.5_(1,4‐NDPAH_2_)], diameter 2.6 Å, suitable for water adsorption.


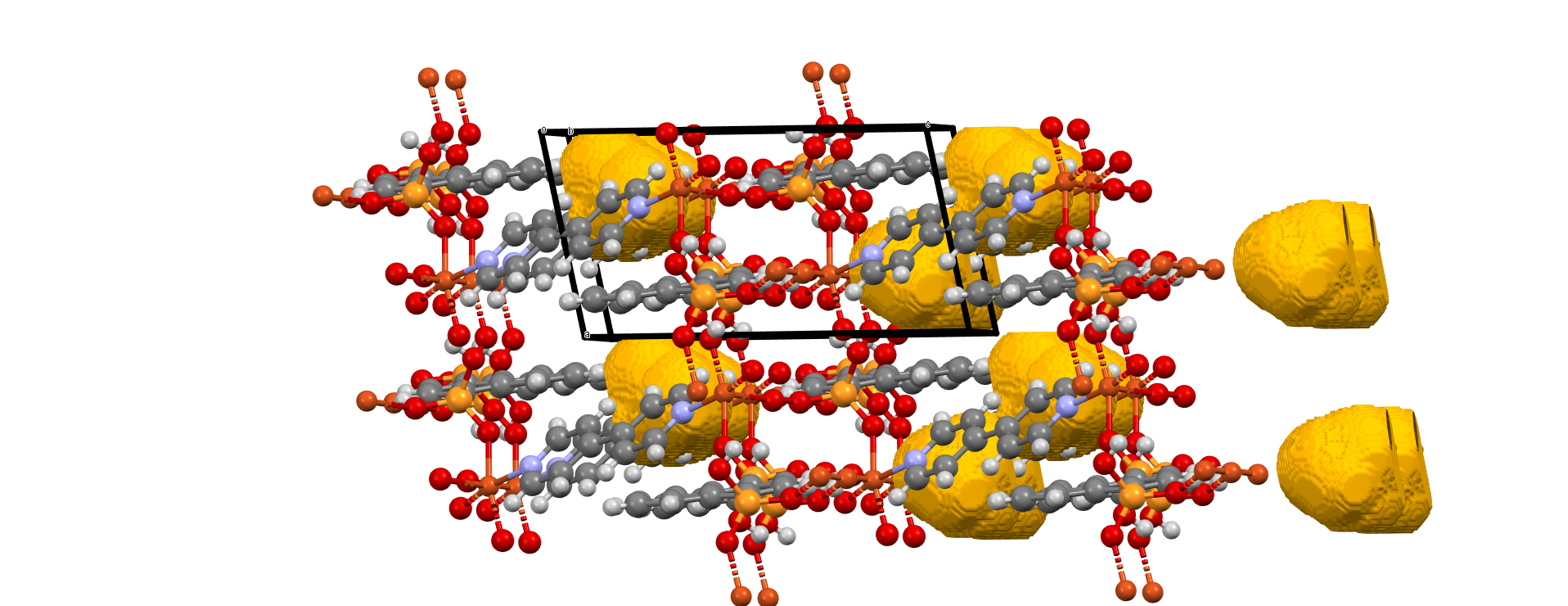


c

a


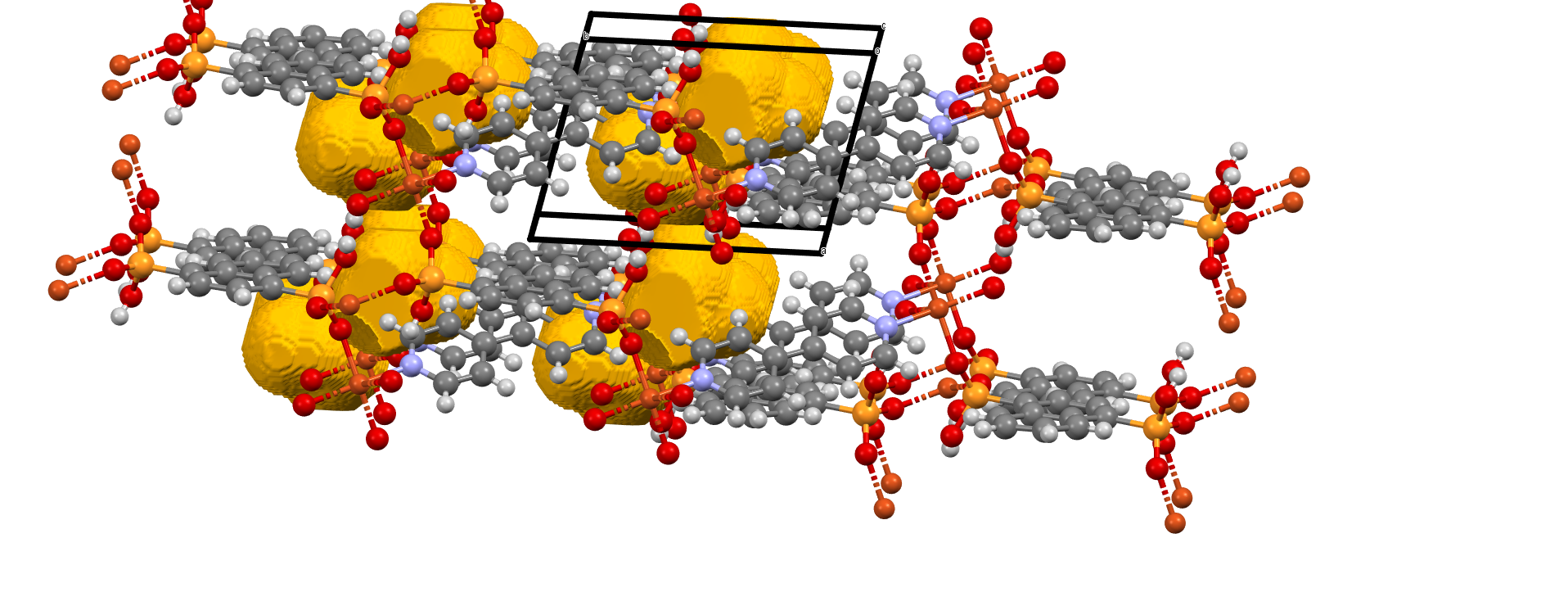


b

a

**Figure S20:** Voids in the MOF [Cu(4,4’‐bpy)_0.5_(1,4‐NDPAH_2_)], diameter 3.2 Å, suitable for CO_2_ adsorption.

1. **Optical spectroscopy**

We measured diffuse reflectance spectra of TUB41 in an integrating sphere (diameter of 120 mm, internally coated with BenFlect® with a reflectance > 99% between 350 nm and 2500 nm) setup on a FLS1000 luminescence spectrometer equipped with a 450 W Xe arc lamp, double grating Czerny-Turner monochromators in both excitation and emission compartment and a thermoelectrically cooled (–20 °C) PMT-980 detector from Hamamatsu. All spectra were corrected for wavelength-dependent grating efficiency, detector sensitivity, and fluctuating lamp intensity. The diffuse reflectance, *R*_∞_, was converted to the Kubelka-Munk function, *K*/*S*, given by equation (1), which is proportional to the effective absorbance, *A*, of the powdered sample,

$\frac{K}{S}=f\left( R_{\infty} \right)=\frac{(1-R_{\infty})^{2}}{2R_{\infty}}\propto A$ (1)

**
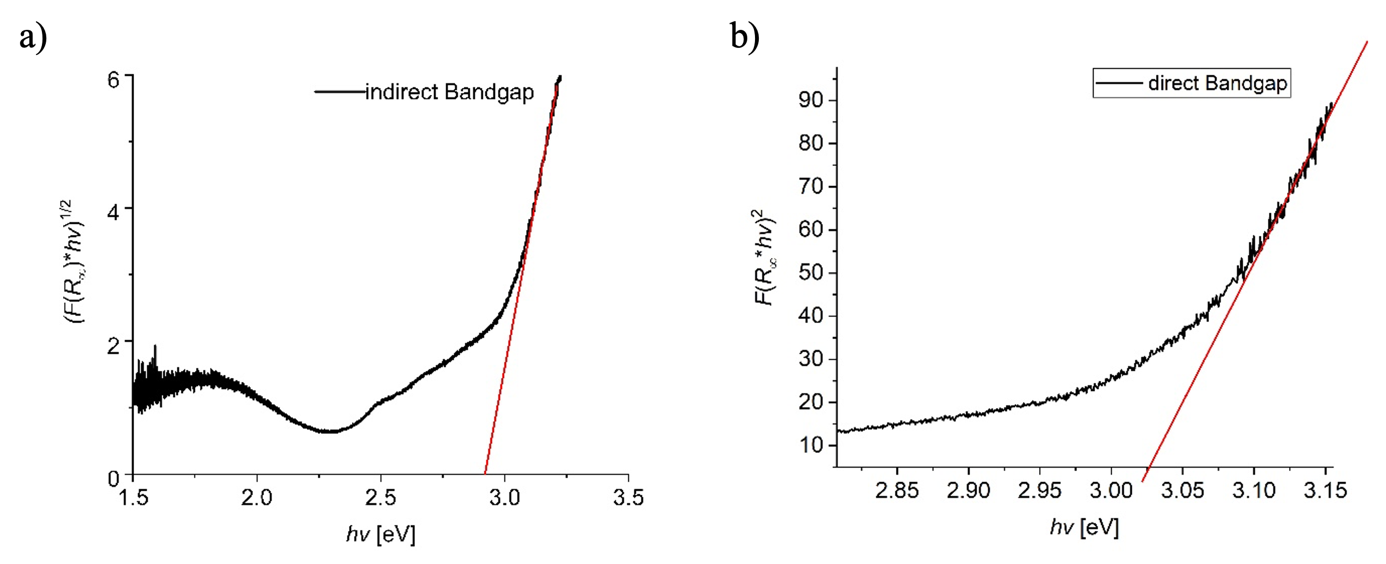
**if the thickness of the powder slab is sufficiently high such that transmittance along the layer is negligible. Tauc plots were generated by plotting (*f*(*R*_∞_) ∙ *E*)^1/2^ vs. incident photon energy *E* = *h*𝜈 (with *h* = 6.626 ∙ 10^-34^ Js as Planck’s constant and 𝜈 as the frequency) for an indirect band gap and (*f*(*R*_∞_) ∙ *E*)^2^ vs. incident energy *E* for a direct band gap.

**Figure S21**: Tauc plots reflecting the a) indirect and b) direct band gap of TUB41.

1. **SEM images**

Scanning electron microscopy (SEM) images and element mapping analysis were recorded on a Jeol JSM-6510LV QSEM electron microscope equipped with a LaB6 filament and a Bruker XFlash 410-M EDX detector at an acceleration voltage of 20 kV. Prior to the measurement, samples were coated with gold using a Jeol JFC 1200 sputter coater.

1. **In-situ variable temperature powder x-ray diffraction (VT-PXRD)**

VT-XRD and isothermal XRD experiments were done using Rigaku Smartlab X-ray Diffractometer equipped with Cu K_α_ x-ray source (λ = 1.54059 Å) with a Hypix-3000 detector, measuring in 1D scanning mode with Bragg–Brentano geometry in horizontal position. The powder sample was prepared on a corundum sample holder and was mounted into a HTK1200N heating stage from Anton Paar. All measurements were performed under normal atmospheric conditions, no vacuum or inert gas was applied. Measurements were performed with an incident slit of 1 mm, 10 mm limiting slit combined with an incident soller slit of 5° and receiving slits #1 of 20 mm and #2 “open”. A K_β_ filter was installed before the detector. Diffraction patterns were collected between 25°C to 350 °C heating rate of 2 K/min resulting in 2 measurements within 5K. For isothermal XRD the target temperature was held for 3.5 min every 5K. Diffraction was measured from 6-24 °2θ with a step size of 0,01 ° and a speed of 10 °/min, resulting in a 3 min 1 s measurement.

1. **Molecular dynamics (MD) simulation**

**Simulation details:**

We adopted the experimentally determined unit cell dimensions and coordinates of the heavy atoms for TUB41, adding hydrogen atoms at appropriate positions. A DFT geometry optimization was subsequently performed in CP2K, employing the PBE functional along with GTH-PBE pseudopotentials, DZVP-MOLOPT-SR-GTH basis set, and DFT-D3(BJ) dispersion corrections. The optimized structure was then used to construct a 12 × 4 × 4 supercell as the initial structure for the MD simulations. Using the this supercell, we built several systems containing varying concentrations of H₂O and CO₂. Specifically, four systems with different H₂O concentrations (containing 10, 30, 100, and 300 H₂O molecules) and three systems with different CO₂ concentrations (containing 10, 30, and 100 CO₂ molecules) were constructed to investigate the distribution, adsorption, and diffusion properties of H₂O and CO₂ within the MOF.

The flexible UFF4MOF force field was employed for TUB41, with the atomic charges fitted using the restrained electrostatic potential–repeating electrostatic potential extracted atomic (RESP-REPEAT) method. The rigid TIP4P model was used for the water molecules, and the TraPPE model was selected for the CO₂ molecules. Interactions between different atom types were determined using the Lorentz–Berthelot mixing rules. A cutoff radius of 12.5 Å was applied for the van der Waals interactions, and the conversion radius between long-range and short-range Coulomb interactions was set to 12 Å. The particle–particle particle–mesh (PPPM) algorithm with a force accuracy set to 1e⁻⁵ was utilized to calculate the long-range electrostatic interactions. Long-range tail corrections were also applied to enhance the accuracy of pressure and energy calculations.

All systems were simulated using LAMMPS (version lammps-omp/20230802). An energy minimization was first performed, followed by a 100 ps NVT equilibration run during which the temperature was gradually increased from 10 K to 300 K. Subsequently, a 50 ns NVT production run was conducted, with a time step of 0.5 fs. Temperature control was achieved using a Nosé–Hoover thermostat. During the production run, the trajectory data was recorded every 1 ps for further analysis. MDAnalysis (version 2.8.0) was used to calculate radial distribution functions (RDFs) and hydrogen bond numbers. In-house Python scripts were used to generate atomic trajectory overlay maps, and LAMMPS was used to compute mean-squared displacements.

**Atomic trajectory overlay maps:**

In all the figures shown below, cyan and green represent the oxygen and hydrogen atoms of H₂O, respectively; red and orange represent the carbon and oxygen atoms of CO₂, respectively; gold, dark blue, dark orange, dark red, black, and gray represent copper, nitrogen, phosphorus, oxygen, carbon, and hydrogen atoms, respectively, in the MOF framework. The *x* and *y* axes of the figures correspond to position, expressed in Ångstroms (Å).


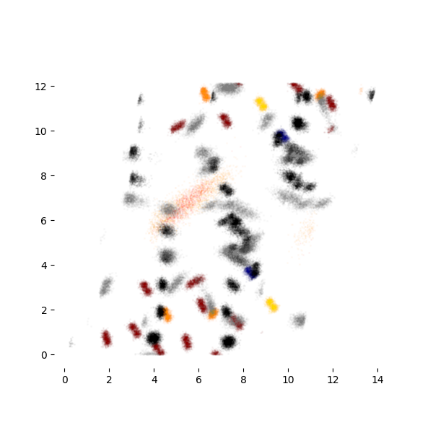

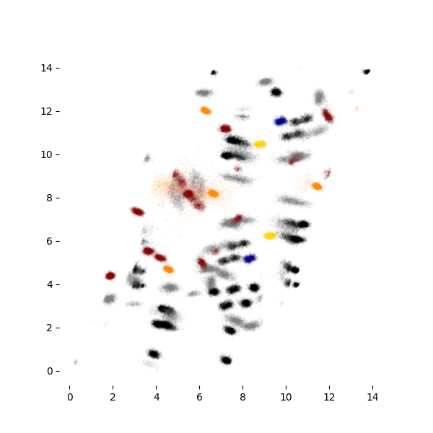

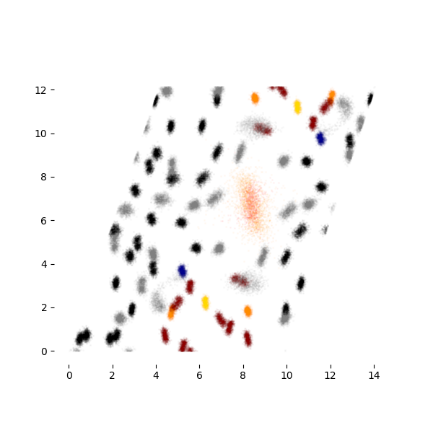


**Figure S22**: Atomic trajectory overlay maps for TUB41 with 10 CO₂ molecules. From left to right: Projections onto a plane perpendicular to the z, y, and x directions.


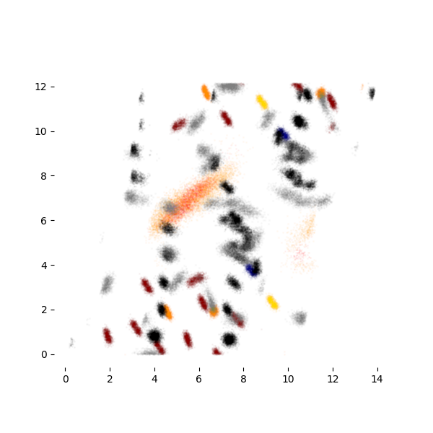

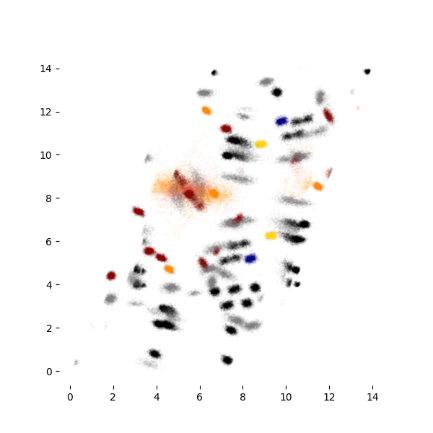

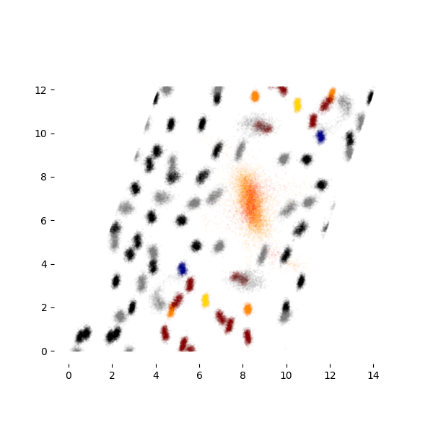


**Figure S23**: Atomic trajectory overlay maps for TUB41 with 30 CO₂ molecules. From left to right: Projections onto a plane perpendicular to the z, y, and x directions.


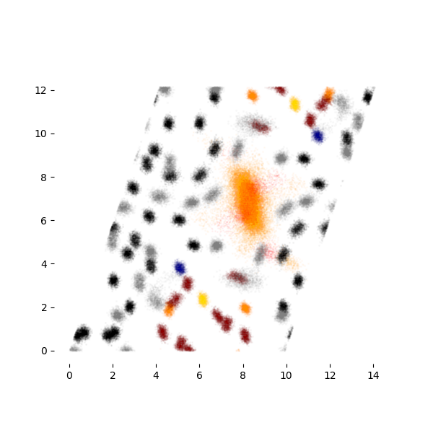

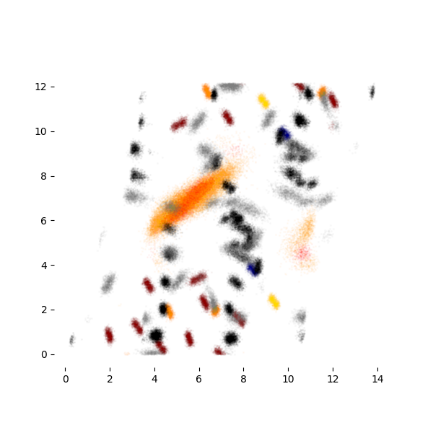

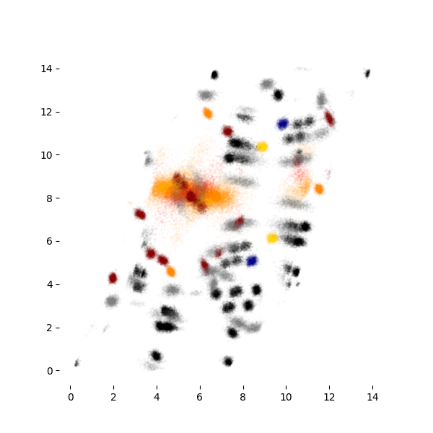


**Figure S24**: Atomic trajectory overlay maps for TUB41 with 100 CO₂ molecules. From left to right: Projections onto a plane perpendicular to the z, y, and x directions.


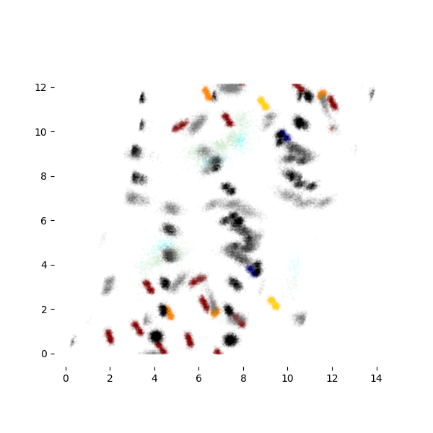

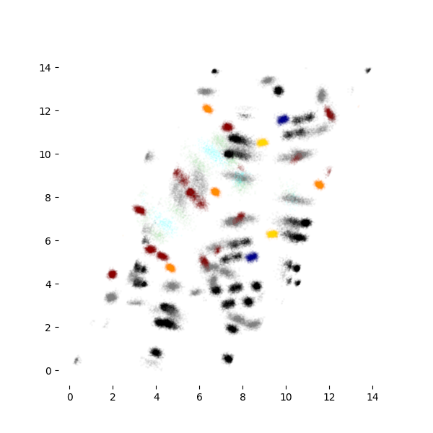

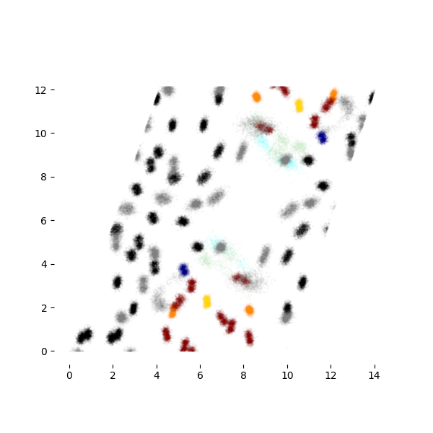


**Figure S25**: Atomic trajectory overlay maps for TUB41 with 10 H₂O molecules. From left to right: Projections onto a plane perpendicular to the z, y, and x directions.


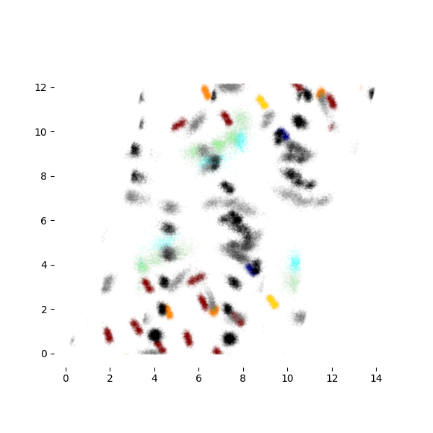

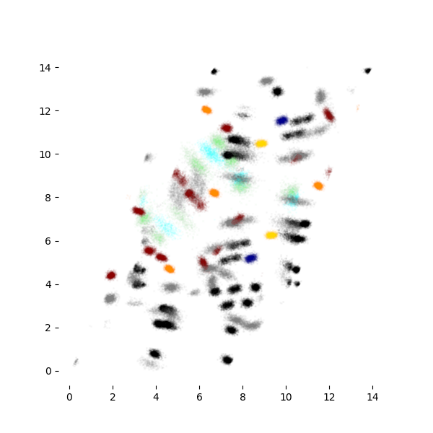

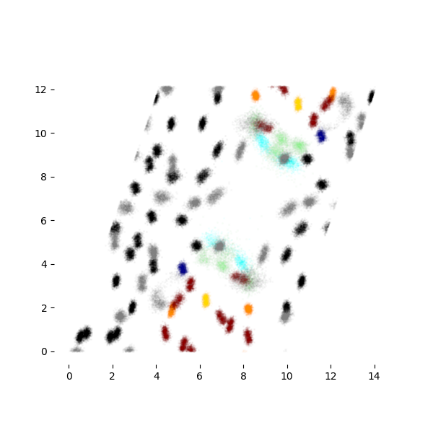


**Figure S26**: Atomic trajectory overlay maps for TUB41 with 30 H₂O molecules. From left to right: Projections onto a plane perpendicular to the z, y, and x directions.


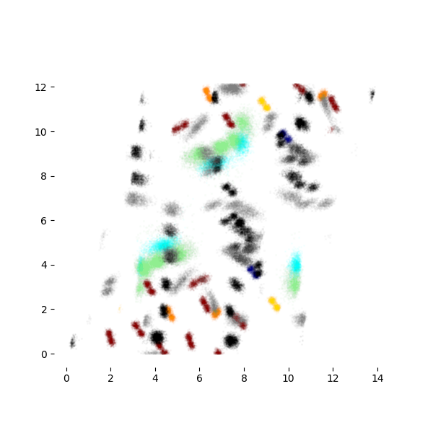

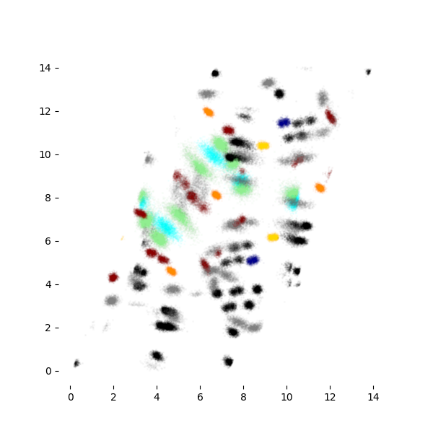

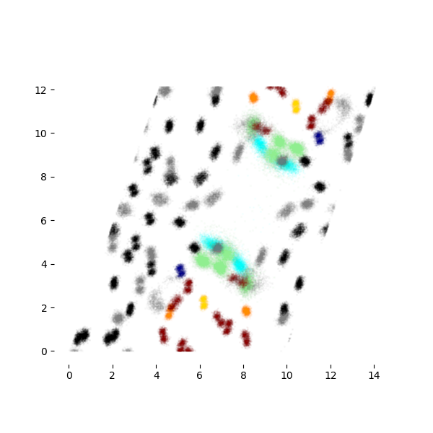


**Figure S27**: Atomic trajectory overlay maps for TUB41 with 100 H₂O molecules. From left to right: Projections onto a plane perpendicular to the z, y, and x directions.


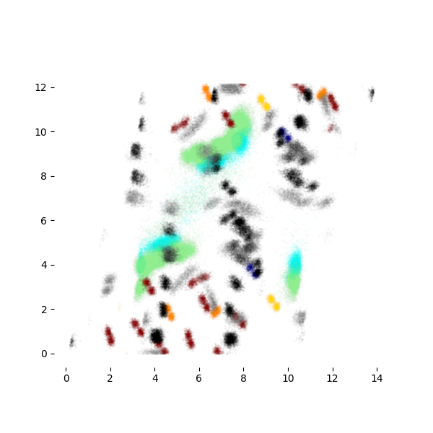

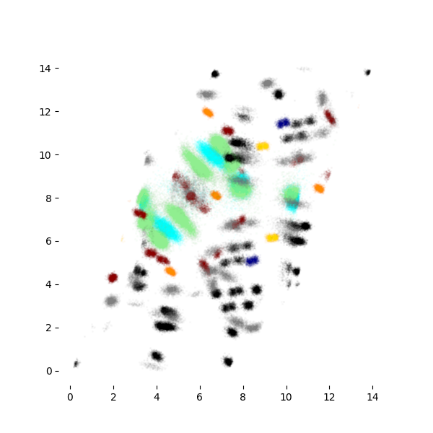

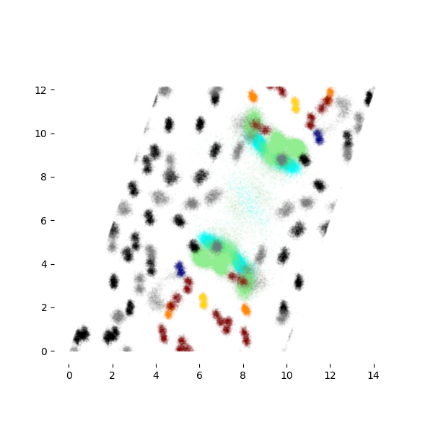


**Figure S28**: Atomic trajectory overlay maps for TUB41 with 300 H₂O molecules. From left to right: Projections onto a plane perpendicular to the z, y, and x directions.

**RDF plots:**

a) b)


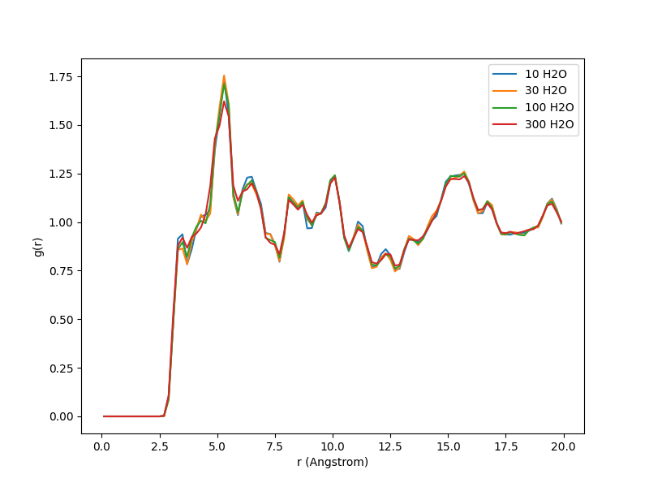

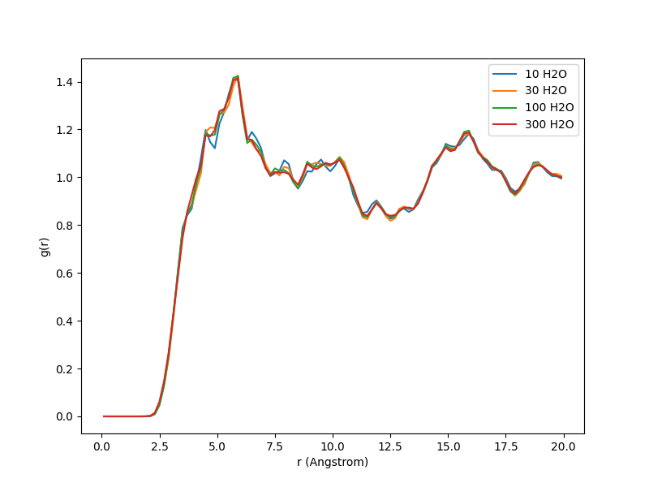


c) d)


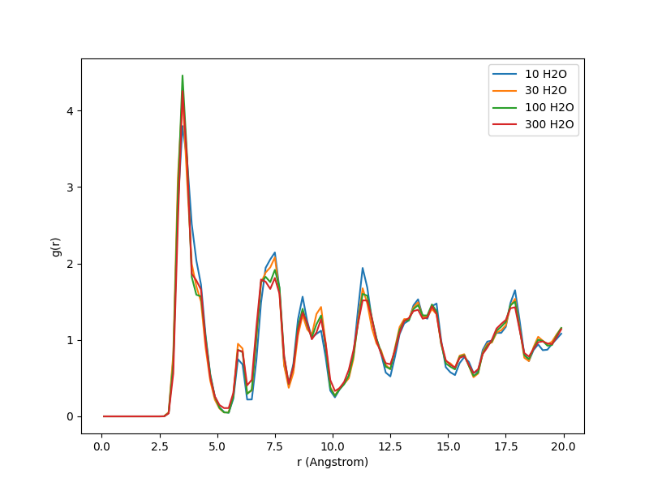

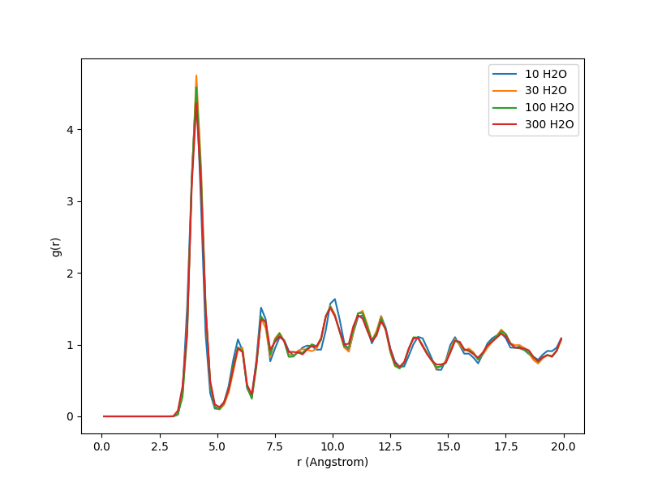


e) f)


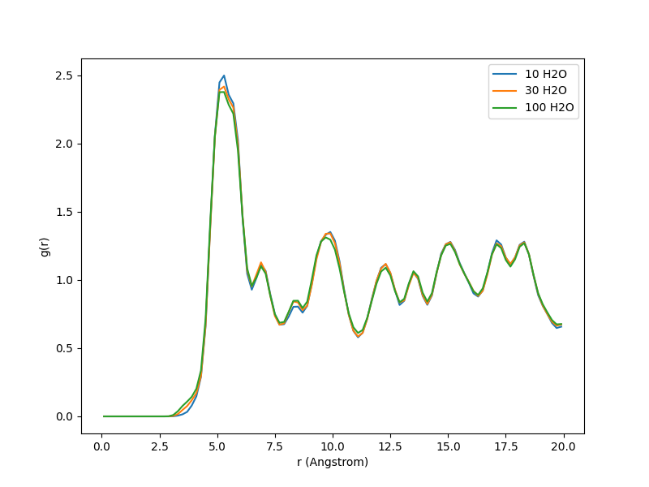

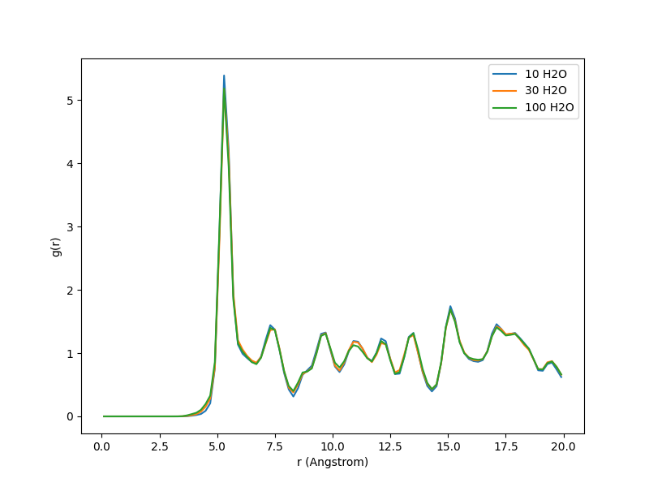


**Figure S29:** RDFs between atoms in CO_2_/H_2_O and atoms in the TUB41 framework for all systems. a) O (H_2_O) – C (framework) b) H (H_2_O) – C (framework) c) O (H_2_O) – (framework) d) O (H_2_O) – P (framework) e) O (CO_2_) – C (framework) f) C (CO_2_) – C (framework).


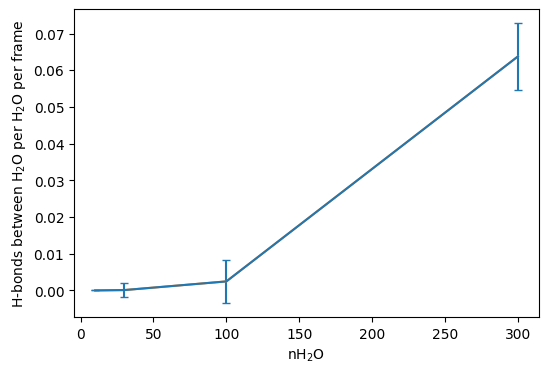


**Figure S30:** Average number of hydrogen bonds between H_2_O molecules for the water-containing systems, normalized by both the number of trajectory frames and the number of H_2_O molecules.
